# Increased Akt-Driven Glycolysis Is the Basis for the Higher Potency of CD137L-DCs

**DOI:** 10.3389/fimmu.2019.00868

**Published:** 2019-04-24

**Authors:** Qun Zeng, Karthik Mallilankaraman, Herbert Schwarz

**Affiliations:** ^1^Department of Physiology, Yong Loo Lin School of Medicine, National University of Singapore, Singapore, Singapore; ^2^Immunology Programme, Life Sciences Institute, National University of Singapore, Singapore, Singapore

**Keywords:** CD137L-DC, metabolism, glycolysis, Akt, mTOR, lipid synthesis, succinate

## Abstract

CD137 ligand-induced dendritic cells (CD137L-DCs) are a new type of dendritic cells (DCs) that induce strong cytotoxic T cell responses. Investigating the metabolic activity as a potential contributing factor for their potency, we find a significantly higher rate of glycolysis in CD137L-DCs than in granulocyte macrophage colony-stimulating factor (GM-CSF) and interleukin 4 induced monocyte-derived DCs (moDCs). Using unbiased screening, Akt-mTORC1 activity was found to be significantly higher throughout the differentiation and maturation of CD137L-DCs than that of moDCs. Furthermore, this higher activity of the Akt-mTORC1 pathway is responsible for the significantly higher glycolysis rate in CD137L-DCs than in moDCs. Inhibition of Akt during maturation or inhibition of glycolysis during and after maturation resulted in suppression of inflammatory DCs, with mature CD137L-DCs being the most affected ones. mTORC1, instead, was indispensable for the differentiation of both CD137L-DCs and moDCs. In contrast to its role in supporting lipid synthesis in murine bone marrow-derived DCs (BMDCs), the higher glycolysis rate in CD137L-DCs does not lead to a higher lipid content but rather to an accumulation of succinate and serine. These data demonstrate that the increased Akt-driven glycolysis underlies the higher activity of CD137L-DCs.

## Introduction

With the recent success of immune checkpoint inhibitors and chimeric antigen receptor T cells (CAR-T), tumor immunotherapy finally had its long-awaited breakthroughs. However, there are many cancer types where these two approaches have low to no efficacy (1–3). Examples would be solid cancers that lack a cell surface tumor associated antigen (TAA) that can be targeted by CAR-T, and cancers that failed to induce an immune response (2, 3).

Dendritic Cells (DCs) as the pivotal link between the innate immune and the adaptive immune system have been the focus of immunological researches for the last several decades. DC-based immunotherapy for cancer has been proven safe and to prolong survival but the clinical response and efficacy are disappointing (4). To date most of the DCs for cancer immunotherapy are generated by treating patients' monocytes with granulocyte macrophage colony-stimulating factor (GM-CSF) and interleukin 4 (IL-4) (4, 5), which are generally referred to as monocyte-derived DCs (moDCs). We have discovered a new type of DCs, CD137L-DCs, which are derived from monocytes by CD137 ligand (CD137L) reverse signaling (6). CD137L-DCs are only found in human but not in mouse because of the difference in human and mouse CD137L (7). Nevertheless, CD137L-DCs are more potent than moDCs in stimulating cytotoxic T cells in an antigen-specific manner and driving a T helper 1 type response (8). T cells activated by CD137L-DCs are less exhausted and metabolically more active (9). CD137L-DCs are promising candidates for the still unmet need of an effective immunotherapy for many types of cancer. A clinical trial testing the safety and optimal dose of CD137L-DCs for the treatment of nasopharyngeal carcinoma is currently ongoing (NCT03282617).

There is accumulating evidence that metabolic reprograming underpins the transition of immune cells between the quiescent and the activated state. The same cells activated by different stimuli usually induce distinctive metabolic programs and the metabolism in turn influences the fate of the cell development. This mutual regulation is particularly evident during T cell differentiation (10) and macrophage polarization (11). DCs are a heterogeneous population consisting of different subsets (12). However, because of the rarity of DCs in peripheral blood, the knowledge of DC metabolism is mainly gained from murine bone marrow-derived DCs (BMDCs). During the activation of BMDCs by toll like receptor (TLR) ligands, especially the TLR4 ligand LPS, BMDCs switch from oxidative phosphorylation (OXPHOS) to glycolysis. This shift is executed in two different stages: The early increase of glycolysis is inducible nitric oxide synthase (iNOS)-independent and mediated by TBK1-IKKε-Akt, while the latter long-term commitment to glycolysis is PI3K-Akt-mTOR-mediated and dependent on iNOS, which generates NO to suppress OXPHOS (13). Glycogenolysis also contributes to the early glycolytic burst in both LPS-activated human moDCs and in murine BMDCs (14). Unlike tumor cells and T cells that rely on glycolysis to provide intermediates as building blocks for proliferation, non-proliferative BMDCs utilize glycolysis mainly to provide acetyl-CoA and nicotinamide adenine dinucleotide phosphate (NADPH) for the synthesis of lipids, leading to an expansion of endoplasmic reticulum (ER) and the Golgi apparatus and increased synthesis and transport of proteins for DC activation (15).

Nevertheless, one should be cautious in applying findings obtained in murine BMDCs to the other types of DCs, as notable differences in metabolism have been found between different subsets of DCs (16). For example, iNOS is induced in LPS-activated murine BMDCs but not the murine classical DCs isolated from the spleen (17). Furthermore, most clinical trials on moDCs to date use a cocktail of cytokines instead of LPS to mature moDCs (4). Whether CD137L-DCs and moDCs matured by cytokine cocktails share similar metabolism as murine BMDCs is unknown. In this study, we have compared the metabolism of CD137L-DCs with that of moDCs, characterized the metabolism-regulating signaling pathways, and explained the high potency of CD137L-DCs from a metabolic perspective. We find that CD137L-DCs are characterized by high Akt-driven glycolysis that is important for both the activation of CD137L-DCs and the persistence of their activated state.

## Materials and Methods

### Antibodies and Inhibitors

Antibodies to the following proteins were purchased from the indicated vendors: mouse IgG1 Kappa (clone MOPC21) Sigma-Aldrich (St. Louis, MO, USA). CD137L (clone 5F4) Biolegend (San Diego, CA, USA). CD3 (clone OKT3), CD40 (clone 5C3) and PD-L1 (clone M1H1) Affymetrix eBioscience (San Diego, CA, USA). CD80 (clone 2D10), CD86 (clone IT2.2) and CD70 (clone 113-16) Biolegend. Phospho-Akt (Ser473) (clone D9E), Pan-Akt (clone 40D4), Phospho-S6 Ribosomal Protein (Ser235/236), S6 Ribosomal Protein (clone 54D2), Phospho-p44/42 MAPK (Erk1/2, Thr202/Tyr204), p44/42 MAPK (Erk1/2, clone L34F12), phospho-AMPKα (Thr172, clone 40H9), AMPKα (clone F6), phospho-GSK-3β (Ser9, clone D85E12), GSK-3β (clone 3D10), rabbit IgG-HRP, mouse IgG-HRP, beta-actin (clone 13E5), and PathScan® Intracellular Signaling Array Kit from Cell Signaling Technology (Danvers, MA, USA). GAPDH (clone 6C5) Abcam (Cambridge, UK). LY294002 and Rapamycin from Cell Signaling Technology. DMSO, 2-DG, C75, and TOFA from Sigma-Aldrich.

### Differentiation of DCs

Human peripheral blood mononuclear cells (PBMCs) were isolated from human blood by Ficoll-Paque (GE Healthcare, Chi, IL, US) density gradient centrifugation. Monocytes were isolated from PBMCs by using the EasySep Human Monocyte Isolation Kit (#19359, StemCell technologies, Vancouver, Canada). Isolated monocytes were cultured in RPMI-1640 supplemented by 10% FBS, 50 μg/ml streptomycin and 50 IU/ml penicillin (R10 PS medium). CD137L-DCs were differentiated by seeding monocytes on anti-CD137L antibody pre-coated plate (5 μg/ml, 4°C overnight) at 1 million/ml for 7 d. moDCs were differentiated by treating monocytes with 100 ng/ml IL-4 and 80 ng/ml GM-CSF (ImmunoTools, Friesoythe, Germany) for 7 d. CD137L-DCs were matured by 1 μg/ml Resiquimod (R848, InvivoGen, San Diego, CA, USA) and 50 ng/ml IFN-γ (#285-IF-100, R&D, Minneapolis, MN, USA) and moDCs were matured by 10 ng/ml IL-6, IL-1β, TNFα (ImmunoTools) and PGE2 (#P0409, Sigma-Aldrich) in the last 18 h of differentiation.

For experiments involving inhibitors, cells were incubated with inhibitors 1 h prior to inducing differentiation or maturation. During differentiation, 2 μM LY294002 and 10 nM Rapamycin were used. After 1 day of DC differentiation, inhibitors were washed out and developing DCs were supplemented again with differentiation cytokines. During maturation, 50 μM 2-DG, 10 μM LY294002, 50 nM Rapamycin, 20 μM C75 and 20 μM TOFA were used.

### Mixed Lymphocyte Reaction

T cells were isolated from PBMCs using the EasySep Human T cell Isolation Kit (#17951, StemCell technologies), and labeled by CellTrace™ Violet dye (#C34557, ThermoFisher Scientific). Allogenic mixed lymphocyte reaction (MLR) was done by co-culturing 2 × 10^4^ DCs generated from one donor with 2 × 10^5^ T cells isolated from another donor in AIM V™ medium (#12055091, ThermoFisher Scientific) supplemented with 2% human AB serum (#H3667, Sigma-Aldrich) for 5 d in 96-well plates. The supernatants were collected for cytokine measurements. The proliferation of T cells was quantified by the dilution of CellTrace™ Violet dye which was measured by flow cytometry after gating for CD3^+^ cells.

### Seahorse Metabolic Assays

Seahorse XFe24 FluxPaks, XF Base Medium Minimal DMEM (0 mM Glucose), Seahorse XF Glycolysis Stress Test Kit, XF Mito Fuel Flex Test kit and XF Cell Mito Stress Test Kit were purchased from Agilent (Santa Clara, California, USA). The characterization of DC metabolism was done as described previously (18). Briefly, harvested DCs were washed with PBS once, resuspended in assay medium to make 2 million/ml (1~4 million/ml), and 0.1 ml DCs were seeded per well in poly-D-lysine (#P6407, Sigma-Aldrich) coated plate. DCs were equilibrated in CO_2_-free incubator at 37°C for 30 min. After the medium was topped up to 0.5 ml, the plate was equilibrated in CO_2_-free incubator at 37°C for another 30 min before being loaded into the machine. The final concentrations of drugs were: 10 mM Glucose, 1 μM Oligomycin, 50 mM 2-DG, 3 μM FCCP, 1 μM Rotenone+ 1 μM Antimycin A, 3 μM BPTES, 4 μM Etomoxir, and 2 μM UK5099.

### Western Blot

For PathScan® Intracellular Signaling Array experiments, cell lysates were prepared and incubated according to the protocol. The signal were measured by a ChemiDoc (Biorad, CA, USA) machine. For LY294002 or Rapamycin treatment, cells were pretreated with the inhibitors for 1 h before maturation or differentiation. 1 or 2 h afterwards, cells were washed with ice-cold PBS twice and lysed by RIPA buffer (#9806, CST) supplemented with protease and phosphatase inhibitor cocktail (#78440, ThermoFisher Scientific) on ice for 10 min. Cell lysates were collected, sonicated by a water bath sonicator, and pelleted at maximum speed at 4°C for 15 min on a bench top centrifuge. The concentrations of cell supernatants were quantified by Bradford assay. Equal amount of proteins was run on a SDS-PAGE gel, transferred to a PVDF membrane and blocked by 5% non-fat milk at room temperature for 1 h. The PVDF membrane was probed with primary antibodies at 4°C overnight, washed with 1% TBST three times, and probed with secondary antibodies at rt. for 1 h. The PVDF membrane was washed again with 1% TBST three times before development. The developed X-ray films were scanned and the bands were semi-quantified by ImageJ.

### qPCR

Total DNA were extracted by organic solvents (19). The mitochondrial DNA copy number per cell was quantified by the ratio of the copy number of mitochondrial tRNA to the copy number of β-2-microglobulin (β2M) (quantified by qPCR) as previously described (20).

### TMRE Staining

CD137L-DCs were grown on cell-culture treated coverslips (#174985, ThermoFisher Scientific). DCs were generated and loaded with TMRE (Δψ_m_ indicator; 100 nM) in the dark for 20 min at 37°C. Cells were then washed and resuspended in Hank's buffered salt solution (HBSS), pH 7.2. Images were acquired using an Olympus IX73 fluorescent imaging system with excitation at 561 nm. Twenty images were collected randomly for each sample, and the fluorescence was quantified using Image J software.

### ELISA

IL-8, IL-10, TNFα, and IL-1β in the supernatant were measured by respective Ready-SET-Go!® Set (eBioscience) ELISA kits according to the protocol. IL-12 and IFN-γ in the supernatant were measured by respective DuoSet ELISA kit according to the protocol (R&D Systems, Minneapolis, USA). All cytokines are in pg/ml.

### Flow Cytometry

CD137L-DCs were washed with cold PBS, incubated in L7^TM^ hPSC Passaging Solution (#FP-5013, Lonza, Basel, Switzerland) at 37°C for 15 min followed by R10 PS medium addition, and harvested by scraping. moDCs were harvested by flushing. For the proper comparison of cell surface markers, moDCs were also incubated with L7^TM^ hPSC Passaging Solution at 37°C for 15 min. Cells were pelleted and washed with cold PBS for once, followed by cell surface Fc receptor blockage by FcR blocking reagent (#130-059-901, Miltenyi Biotec, Bergisch Gladbach, Germany). Cell surface markers were stained at 4°C for 30 min. Cells were spun down and washed with cold FACS buffer twice before the analysis on LSR Fottessa or X20 (BD, NJ, USA) or Attune NxT Flow Cytometer (ThermoFisher Scientific, Carlsbad, CA, USA). For live / dead cell staining, 1 μg/ml 7-AAD (Biolegend) was added 5 min before the measurement by flow cytometry. Data were analyzed with FlowJo 10.

### Lipid Staining

HCS LipidTOX™ Phospholipidosis and Steatosis Detection Kit (#H34158, ThermoFisher Scientific) or BODIPY (#790389, Sigma-Aldrich) was used to stain the lipid. LipidTOX Red phospholipid stain was added to the cell culture 18 h before harvesting. Harvested DCs were washed with PBS and stained with LipidTOX Green neutral lipid stain at room temperature for 30 min. DCs were spun down and washed with PBS once before acquisition on flow cytometer. If BODIPY was used, harvested DCs were stained in the same way as LipidTOX Green neutral lipid stain.

### Metabolomics

The metabolic profiling of organic acids, amino acids, and glycolysis intermediates was done in collaboration with the Duke-NUS metabolomics facility. DCs were washed with ice-cold PBS thrice and resuspended in 50% acetonitrile, 0.3% formic acid. The extraction and measurement of metabolites by LC-MS was done as described previously (21, 22). The concentration of metabolites was normalized by the protein contents of DCs.

### Gene Set Enrichment Analysis (GSEA)

The dataset is obtained from Gene Expression Omnibus, accession number GSE60199 that was deposited by Harfuddin et al. (23). The GSEA analysis was performed by using the javaGSEA Desktop Application (24, 25). For all gene sets, 1,000 permutations with “phenotype” algorithm were used.

### Statistical Analysis

Statistical significance was determined by two-tailed unpaired Student's *t*-test unless specified otherwise. If the sample was normalized by the control, statistical significance was determined by one-sample *t*-test against one. The scatter dot plots and bar charts were plotted by GraphPad Prism 6.

## Results

### CD137L-DCs Have Higher Glycolysis Rates and Akt-mTOR1 Activity

**Figure 1:**
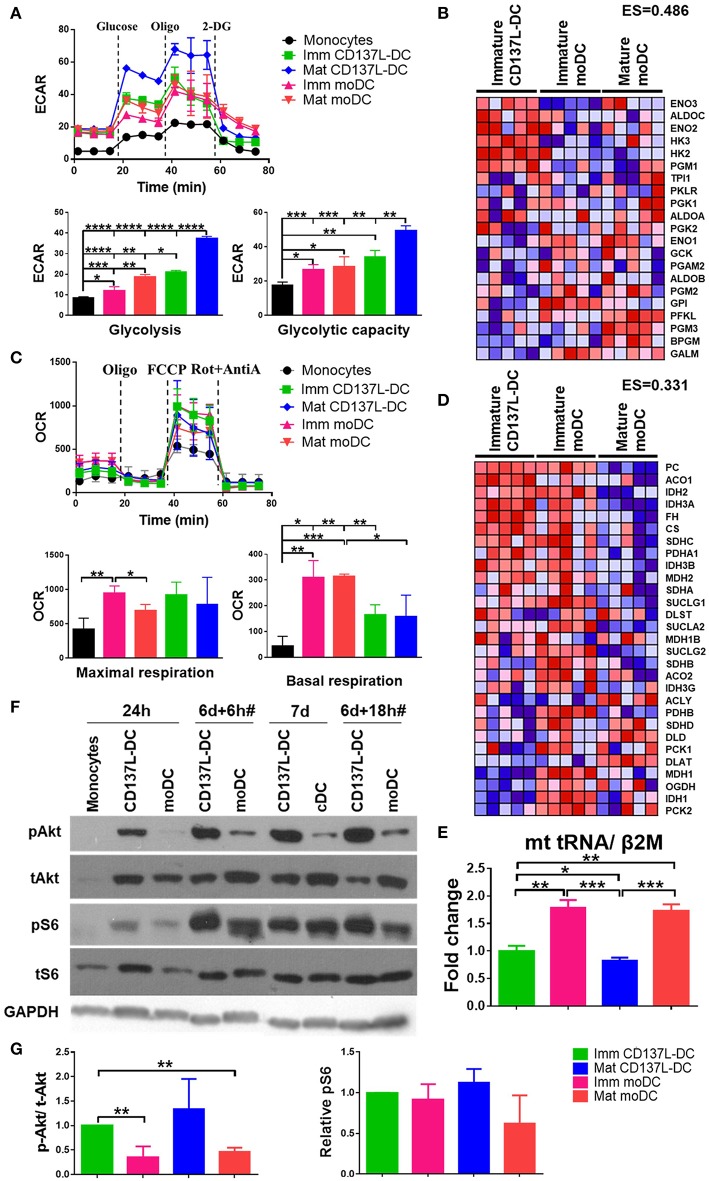
CD137L-DCs have a higher glycolysis rate and higher Akt and mTORC activity than moDCs. **(A)** Glycolysis stress assay and **(C)** Mitostress assay were done by Seahorse XFe24 Analyzer. ECAR (pmol/min/Norm. Unit) and OCR (mpH/min/Norm. Unit) were normalized by the cell protein content. The basal glycolysis, glycolytic capacity, basal respiration, and maximal respiration were calculated according to the instructions provided by the kit's manufacturer. The heatmaps of the glycolysis gene signature **(B)** and the TCA gene signature **(D)** were drawn by comparing the levels of RNAs between immature CD137L-DCs and the other DC types (immature moDCs and mature moDCs) by GSEA. Shown are results from five different donors. Red: relatively enriched. Blue: relatively decreased. The enrichment score (ES) of the gene set in immature CD137L-DC relative to immature moDC and mature moDC as a whole is stated above the heatmap. **(E)** Relative mitochondrial counts in different DC types were measured by the mitochondrial DNA copy number. Shown are means ± standard deviations of triplicate measurements. **(F)** Monocytes were differentiated by GM-CSF + IL-4 or anti-CD137L antibody (clone 5F4) or the isotype antibody (clone MOPC-21) for 7 d. Cells were lysed at indicated time points. CD137L-DCs were matured by 1 μg/ml R848 + 50 ng/ml IFN-γ and moDCs were matured by 10 ng/ml IL-6, IL-1β, TNFα, and PGE2 during the last 18 h of differentiation, which is indicated by #. The activation of Akt and ribosome protein S6 were measured by Western blot analysis. These data are representative of three independent experiments. **(G)** The activation of Akt (p-Akt/ t-Akt) and mTORC1 (p-S6/ loading controls) was semi-quantified by ImageJ and normalized by the protein level in immature CD137L-DCs. Data from three different donors were statistically analyzed. ^*^p < 0.05, ^**^p < 0.01, ^***^p < 0.001, ^****^p < 0.0001 (two-tailed, two sample student *t* test).

As the activation of DCs is accompanied by metabolic reprograming to a higher rate of glycolysis (15, 26), we compared the glycolysis rates of CD137L-DCs and moDCs at baseline and under metabolic stress induced by Oligomycin. As expected, all DCs had higher extracellular acidification rate (ECAR) due to higher basal glycolysis rates and glycolytic capacities than the undifferentiated monocytes (Figure 1A). Maturation of both CD137L-DCs and moDCs further elevated basal glycolysis rates. Notably, immature CD137L-DCs have significantly higher basal glycolysis than both immature and mature moDCs, while mature CD137L-DCs have the highest basal glycolysis and glycolytic capacity (Figure 1A). In agreement with the higher glycolysis in CD137L-DCs, GSEA also showed an enrichment in enzymes involved in glycolysis in immature CD137L-DCs (Figure 1B), such as hexokinase 2 (HK2), which is a key enzyme in promoting aerobic glycolysis (27).

As the main source of ATP, mitochondrial respiration has also been studied by measuring the oxygen consumption rate (OCR). After their differentiation from monocytes, all DCs had a higher basal respiration rate and a higher maximal respiration than the starting monocytes, though not all comparisons were statistically significant (Figure 1C), indicating a biogenesis of mitochondria during DC differentiation (28). In line with previous observations (15, 26), moDCs had a lower maximal respiration after maturation. Though the basal respiration in moDCs was higher than in CD137L-DCs, there was no significant difference in maximal respiration between the two types of DCs (Figure 1C), suggesting that the mitochondria in CD137L-DCs are still healthy and that their function is not significantly compromised. In line with their higher basal respiration rate, immature moDCs have a higher enrichment in enzymes involved in the TCA cycle than immature CD137L-DCs and mature moDCs (Figure 1D). The lower basal respiration in CD137L-DCs could be a result of fewer mitochondria than in moDCs (Figure 1E). The average mitochondrial membrane potential, which is controlled by respiration, did not differ significantly among the four types of DCs (Supplementary Figures 1A,B). In fact, the responsiveness of moDCs but not CD137L-DCs to the mitochondrial pyruvate carrier blocker, UK5099, implied that moDCs had a mixed glycolytic and aerobic energy phenotype for glucose utilization while CD137L-DCs were mostly glycolytic (Supplementary Figure 1C).

Signaling pathways mediate and regulate the diverse activities of cells. We utilized the CST PathScan® Intracellular Signaling Array Kit to unbiasedly screen the main signaling pathways for an involvement in CD137L-DC differentiation and maturation. Among the 18 targets screened, the Akt-mTORC1 pathway but not the MAPK or Stat pathways consistently showed a stronger activation in CD137L-DCs than moDCs differentiated from monocytes from two healthy donors (data not shown). This result was further confirmed by Western blot analysis. 24 h after the differentiation was initiated, the nascent CD137L-DCs showed a robust Akt activation that could not be detected in nascent moDCs. Although Akt activation was present in moDCs at later time points, this stronger activation of Akt in CD137L-DCs persisted during the entire period of differentiation and maturation (Figure 1F). Ribosomal protein S6, which is a downstream target of mTORC1, was comparably activated in immature CD137L-DCs and immature moDCs but showed higher activation in mature CD137L-DCs than in mature moDCs (Figure 1F). The result is reproducible with the pooled semi-quantified results shown in Figure 1G. Some comparisons are not statistically significant due to the large donor variation and relatively small sample size of three donors. Other molecules related to mTORC1, such as PRAS40, p70S6, and mTOR itself, also displayed stronger activation in mature CD137L-DCs than in mature moDCs (Supplementary Figure 2).

### Glycolysis Is Essential for Sustaining the Activated State of Mature CD137L-DCs

It has been previously reported that glycolysis is indispensable for the activation of murine BMDCs and human moDCs (15, 26). Our data are in line with these observations. When glycolysis was inhibited by 2-Deoxy-D-glucose (2-DG) during moDCs maturation, expression of CD70 and CD86 was significantly decreased (Supplementary Figure 3A). The maturation of CD137L-DCs was more affected by 2-DG than the maturation of moDCs. For example, CD40, CD70, and IL-12 were downregulated by 2-DG to a much higher extent in mature CD137L-DCs than in mature moDCs (Supplementary Figures 3A,B). This could be explained by the higher rate of glycolysis in mature CD137L-DCs than in mature moDCs.

**Figure 2:**
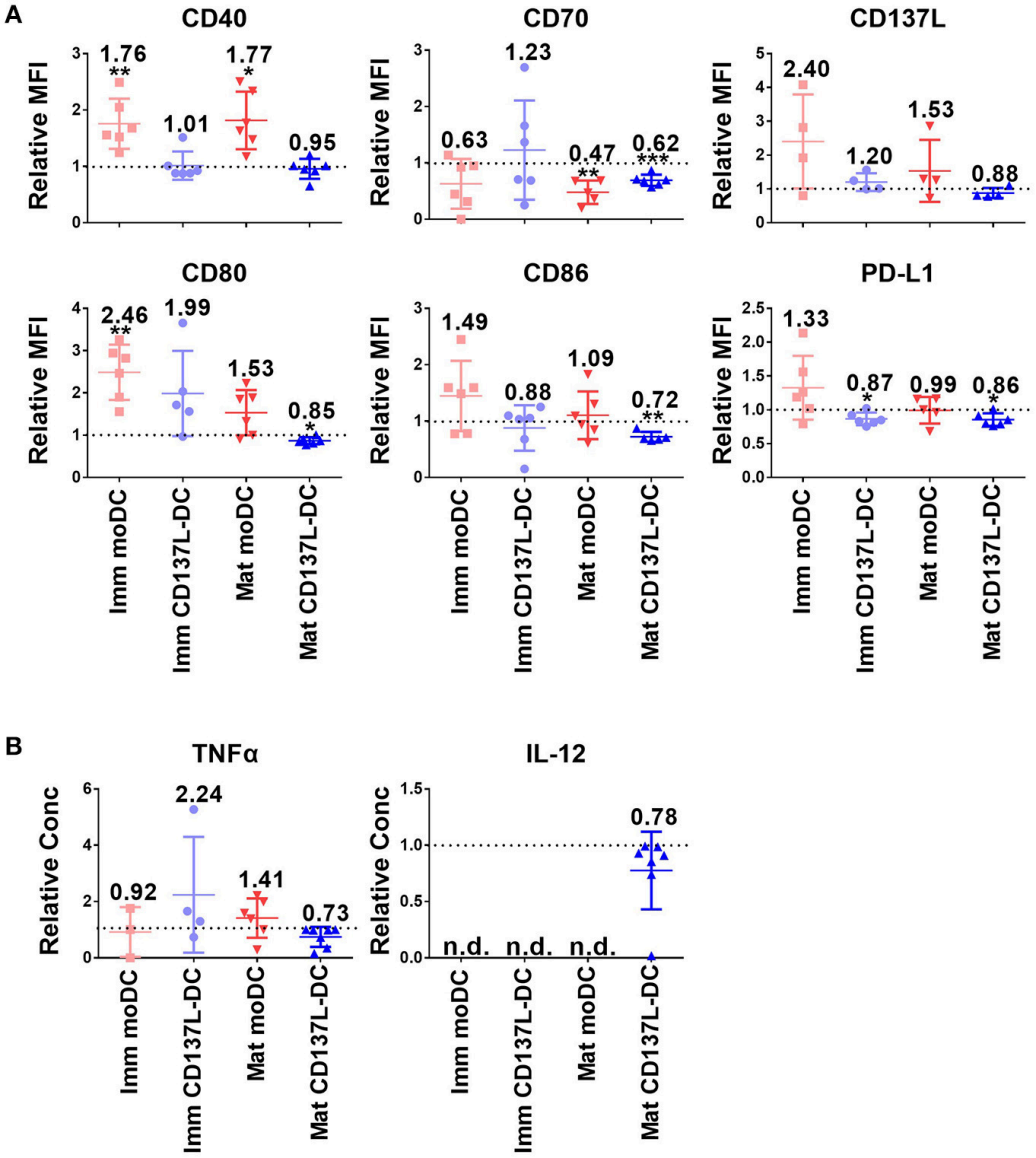
The inhibition of glycolysis after maturation significantly impairs the function of mature CD137L-DCs. DCs were generated and on day 8 treated with 50 mM 2-DG for 24 h. **(A)** Cell surface expression of co-stimulatory and inhibitory molecules was measured by flow cytometry. **(B)** The secretion of cytokines by DCs was measured by ELISA. Depicted are the means ± standard deviations of relative changes upon addition of 2-DG to respective medium controls (values set at 1) from up to six independent experiments with DCs from different donors. ^*^p < 0.05, ^**^p < 0.01, ^***^p < 0.001 (two-tailed, one sample student *t* test). MFI, geometric mean fluorescence intensity. n.d., not detected.

Since DCs used for tumor immunotherapy are always generated in nutrient-rich medium, we investigated how important glycolysis is for the function of different types of *in vitro* generated DCs. Surprisingly, glycolysis remained necessary for the expression of most co-stimulatory molecules examined and for the secretion of inflammatory cytokines even after DC differentiation and maturation had been completed. Representative sets of histogram are shown in Supplementary Figure 4A. Mature CD137L-DCs, which had the highest glycolysis rate, were the DC type most inhibited by 2-DG. For example, the MFI of CD80 decreased in mature CD137L-DC after 2-DG treatment but increased in the other three types of DCs. CD70, CD86, and CD137L also significantly decreased when glycolysis was suppressed by 2-DG (Figures 2A,B). However, this inhibition by 2-DG was not permanent. After 2-DG was washed out and the DCs were cultured in normal medium, all the co-stimulatory molecules increased to the level of control cells (Supplementary Figure 5), indicating that DCs are plastic and responsive to the changes in the environment.

Interestingly, CD137L-DCs treated with 2-DG were more resistant to cell death than moDCs. The inhibition of glycolysis altered the forward scatter and side scatter of mature moDCs but not of mature CD137L-DCs, indicating an increased percentage of cell death in mature moDCs (Supplementary Figure 4B). This vulnerability of mature moDCs to 2-DG induced cell death was further supported by in-plate trypan blue staining (Supplementary Figure 4C), which did not require cell scraping and thereby avoided potential damage to cells. These data tally with the data from the PathScan Intracellular Signaling Array showing that there is more extensive phosphorylation of the Bcl-2-associated death promoter (Bad) and less cleavage of caspase 3 in CD137L-DCs than in moDCs (Supplementary Figure 4D), indicating a lower degree of apoptosis. This dependence of moDCs on glycolysis for cell survival confirms previous findings in murine BMDC (17). In contrast, CD137L-DCs were more viable and not as dependent on glycolysis for cell survival.

### Akt Drives the Increased Glycolysis and Activation of CD137L-DCs

**Figure 3:**
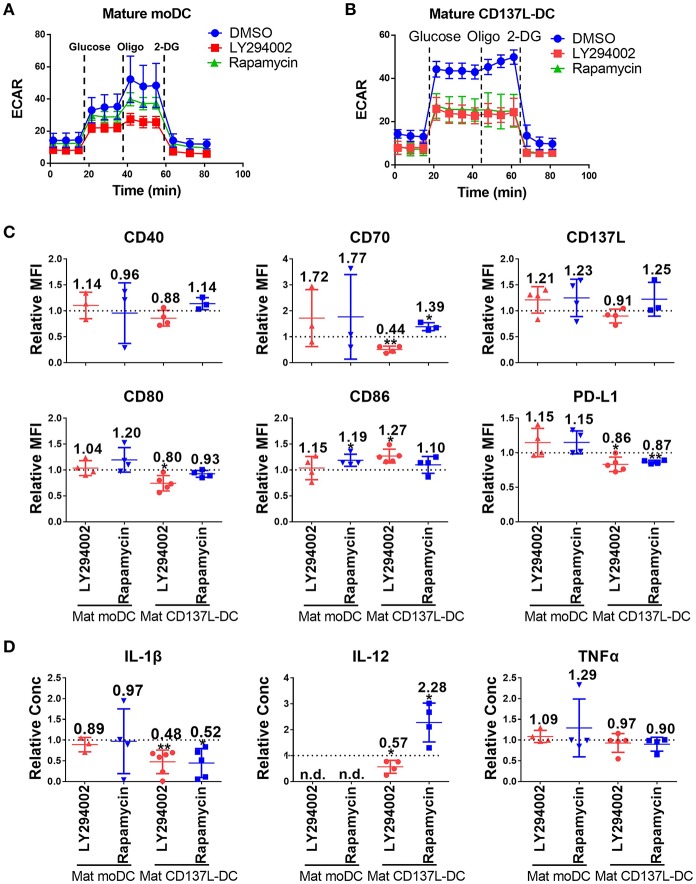
The activation of Akt and mTORC1 is important for the commitment to glycolysis and maturation of CD137L-DCs. DCs were differentiated and pre-treated with DMSO or 10 μM LY294002 or 50 nM Rapamycin for 1 h before maturation. Glycolysis stress assays of **(A)** mature moDCs and **(B)** mature CD137L-DCs were done with a Seahorse XFe24 Analyzer. **(C)** Cell surface expression of co-stimulatory and inhibitory molecules was measured by flow cytometry. **(D)** The secretion of cytokines by DCs was measured by ELISA. Depicted are means ± standard deviations of changes upon addition of 10 μM LY294002 or 50 nM Rapamycin relative to respective DMSO controls (values set at 1) from up to 5 independent experiments with DCs from different donors. ^*^p < 0.05, ^**^p < 0.01 (two-tailed, one sample student *t* test). n.d., not detected.

As demonstrated above, the activation of CD137L-DCs was accompanied by an elevated glycolysis rate and an increased Akt-mTORC1 activity. An increased Akt-mTORC1 activity is the cause of an elevated glycolysis rate in LPS-activated murine BMDCs (26). In order to test whether such a causal relationship is also the case for human DCs, LY294002, an inhibitor of PI3K-Akt, and Rapamycin, an inhibitor of mTORC1, were used. The efficacy and specificity of the inhibitors were first confirmed (Supplementary Figures 6A,B). Inhibition of the Akt-mTORC1 pathway by LY294002 or Rapamycin slightly reduced the increase in glycolysis in mature moDCs (Figure 3A) but completely blocked it in mature CD137L-DCs (Figure 3B). Similarly as the inhibition of glycolysis by 2-DG, inhibition of glycolysis by LY294002 significantly impaired the expression of most co-stimulatory molecules and the secretion of pro-inflammatory cytokines by mature CD137L-DCs, while mature moDCs were not much affected (Figures 3C,D). In contrast, Rapamycin generally increased the expression of co-stimulatory molecules and IL-12 secretion, of which the reason is currently not known.

However, once the DCs were matured, the inhibition of Akt or mTORC1 had little effect on the expression of costimulatory molecules and cytokines by mature CD137L-DCs or mature moDCs (Supplementary Figure 7). The reason for this non-responsiveness may be that the signaling pathways are usually upstream of an activation decision point, and are only active for a short period after encountering a stimulus, such as the TLR ligands or maturation cocktails, while the metabolism is fundamental and active for an extended period.

### mTORC1 Is Indispensable for the Differentiation of CD137L-DCs

**Figure 4:**
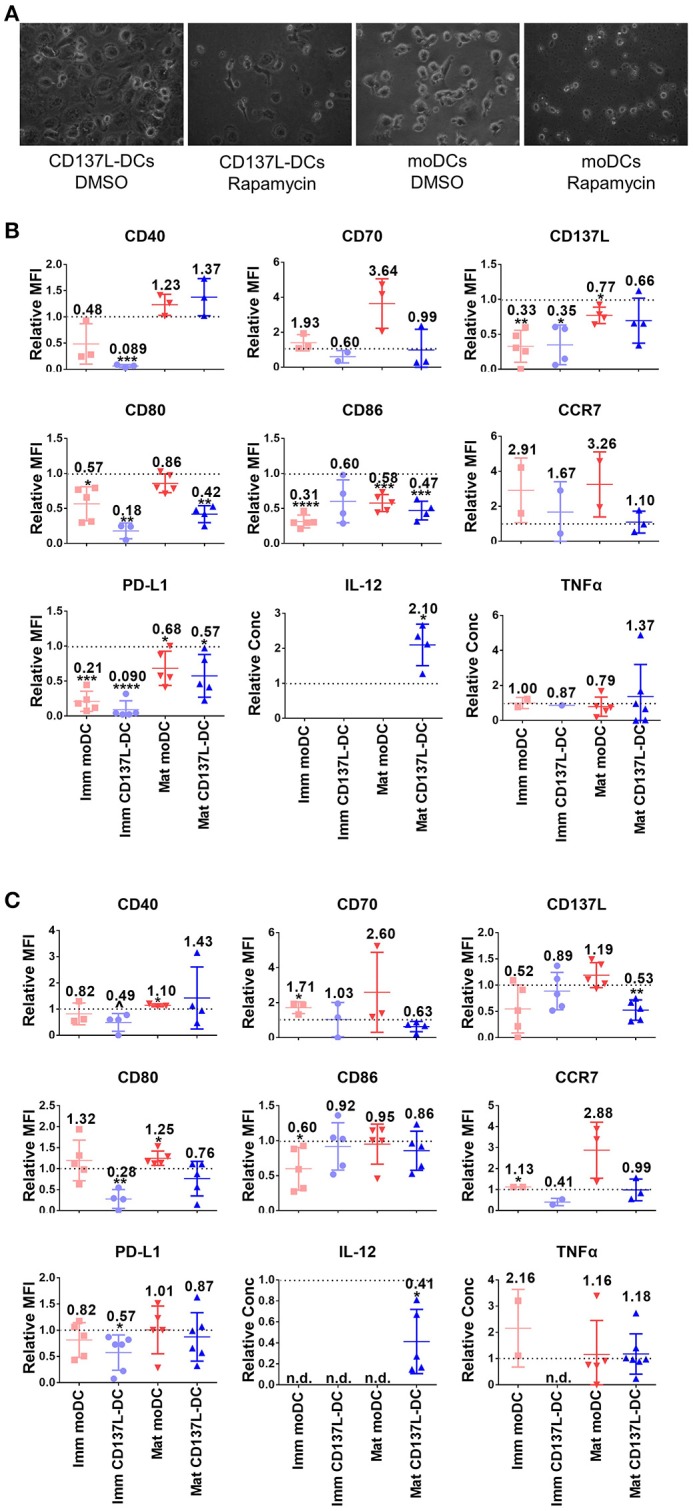
The activation of mTORC1 is indispensable for the differentiation and function of CD137L-DCs and moDCs. Primary monocytes were pre-treated with DMSO or 2 μM LY294002 or 10 nM Rapamycin for 1 h before the differentiation to moDC or CD137L-DCs was induced. 24 h after the initiation of differentiation, inhibitors were washed out and the moDCs were re-supplemented with GM-CSF + IL-4. Where indicated, DCs were matured during the last 18 h of the 7-day culture. **(A)** mTORC1 inhibition by Rapamycin blocks differentiation. Shown are representative photos of DCs, taken before the drug wash-out. Cell surface expression of co-stimulatory and inhibitory molecules and the secretion of cytokines after **(B)** Rapamycin and **(C)** LY294002 treatment were measured. Depicted are means ± standard deviations of changes upon addition of 2 μM LY294002 or 10 nM Rapamycin relative to respective DMSO controls (values set at 1) from up to 7 independent experiments with DCs from different donors. ^*^p < 0.05, ^**^p < 0.01, ^***^p < 0.001, ^****^p < 0.0001, *p* = 0.0584 (two-tailed, one sample student *t* test). n.d., not detected.

Since the PI3K - Akt - mTORC1 pathway was activated early on upon the induction of DC differentiation (Figure 1F), we were wondering whether the PI3K - Akt - mTORC1 pathway could affect the differentiation of monocytes to DCs in addition to its effect on maturation. For that the concentration of inhibitors was first optimized (Supplementary Figures 8A,B). The most striking effect was that inhibition of mTORC1 from 1 h before to 24 h after induction of immature moDCs or CD137L-DCs differentiation always blocked the differentiation of DCs, as evidenced by the absence of the typical morphology of immature moDCs or CD137L-DCs (Figure 4A). After 7 d, fewer live DCs were present. The increased cell death after Rapamycin treatment is mainly a result of differentiation blockade but not of acute cytotoxicity of Rapamycin, since the viabilities of monocytes and nascent moDCs on day 1 were comparable between Rapamycin treatment and the control sample (Supplementary Figure 8C). Analysis of costimulatory molecule expression confirmed that mTORC1 inhibition during differentiation impaired the differentiation of DCs, with immature CD137L-DCs being more affected than immature moDCs (Figure 4B). The effect of Akt inhibition during differentiation was more variable among different donors. Expression of costimulatory molecules and cytokines by immature CD137L-DCs was reduced although the difference was not always statistically significant due to large donor to donor variation (Figure 4C).

The inhibition of Akt or mTORC1 during the first day of differentiation had a long-term influence on DC maturation. IL-12 is usually secreted by activated DCs, especially by mature CD137L-DCs. Even though the inhibitors were washed out by the end of the first day, LY294002-treated mature CD137L-DCs still secreted much less IL-12 while Rapamycin-treated mature CD137L-DCs secreted more IL-12 than the control cells, which is reminiscent of the IL-12 secreted by mature CD137L-DCs treated by LY29002 or Rapamycin during maturation (Figure 3D).

### The Increased Glycolysis During DC Maturation Does Not Fuel Lipid Synthesis

**Figure 5:**
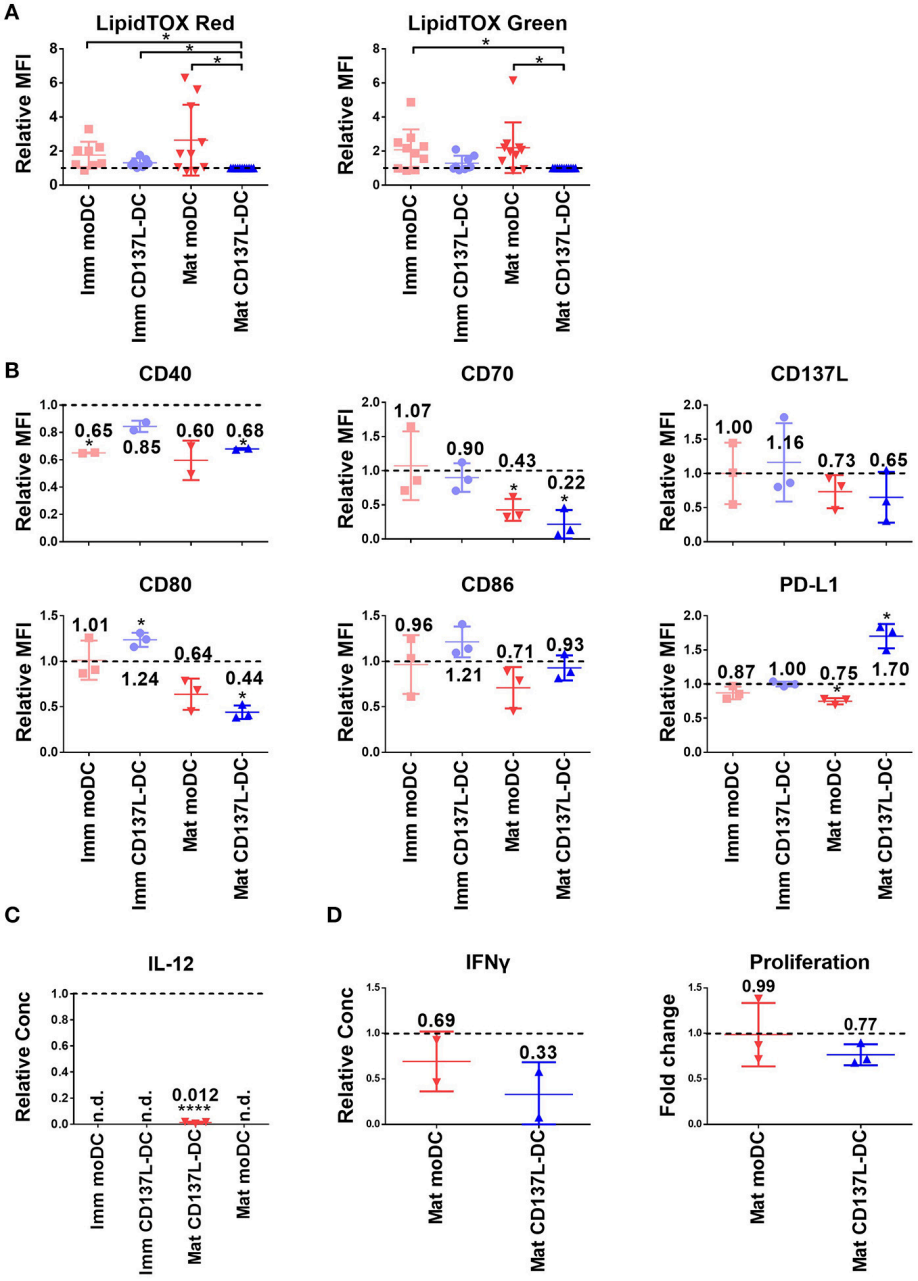
C75 suppresses the maturation of moDCs and CD137L-DCs. **(A)** The phospholipid (left) and neutral lipid (right) contents were measured by LipidTOX reagents. The MFIs of other DCs were normalized by the MFIs of the respective mature CD137L-DCs from up to 11 different donors. DCs were pretreated with 20 μM C75 for 1 h before the last 18 h of maturation/culture. **(B)** Cell surface markers and **(C)** IL-12 in the supernatant were measured. The values were normalized by the respective DMSO-treated controls, and results from three donors were pooled. **(D)** C75-treated DCs were co-cultured with CellTrace Violet labeled allogenic T cells at 1:10 ratio for 5 days. The proliferation of T cells and secretion of IFN-γ by T cells were measured. ^*^p < 0.05, ^****^p < 0.0001 (two-tailed, one sample student *t* test). n.d., not detected.

Glycolysis can favor the function of DCs in many different ways, such as providing carbons and reducing power for lipid synthesis (15). However, there are conflicting data concerning the effect of fatty acid synthesis blockade on DC function (15, 29). Our previous data showed an enrichment in gene expression related to the lipid metabolism in immature CD137L-DCs compared to moDCs (23). But neither had mature CD137L-DCs more phospholipids or neutral lipids than mature moDCs, nor did the lipid content in CD137L-DCs increase upon maturation (Figure 5A), indicating that the synthesis of fatty acids is not the main output of the increased glycolysis in CD137L-DCs.

Since acetyl-CoA carboxylase (ACC) and fatty acid synthase (FASN) are key enzymes for lipid metabolism, we inhibited them with TOFA and C75, respectively. Both inhibitors did not lead to a decrease of the lipid content in treated DCs (data not shown). Nevertheless, C75 significantly suppressed the maturation of CD137L-DCs and moDCs as evidenced by the lower expression of most co-stimulatory molecules (Figure 5B) and the almost complete block of IL-12 secretion (Figure 5C). In the allogenic mixed leukocyte reaction (MLR), T cells activated by C75-treated DCs secreted less IFN-γ and proliferated less than T cells activated by the control DCs (Figure 5D). However, the expression of co-stimulatory molecules and IL-12 were not suppressed by TOFA (Supplementary Figures 9A,B). TOFA-treated DCs did not have a defect in stimulating the T cells (Supplementary Figure 9C). A representative set of histogram of T cell proliferation is shown in Supplementary Figure 9D.

### Succinate and Serine Are Enriched in CD137L-DCs

**Figure 6:**
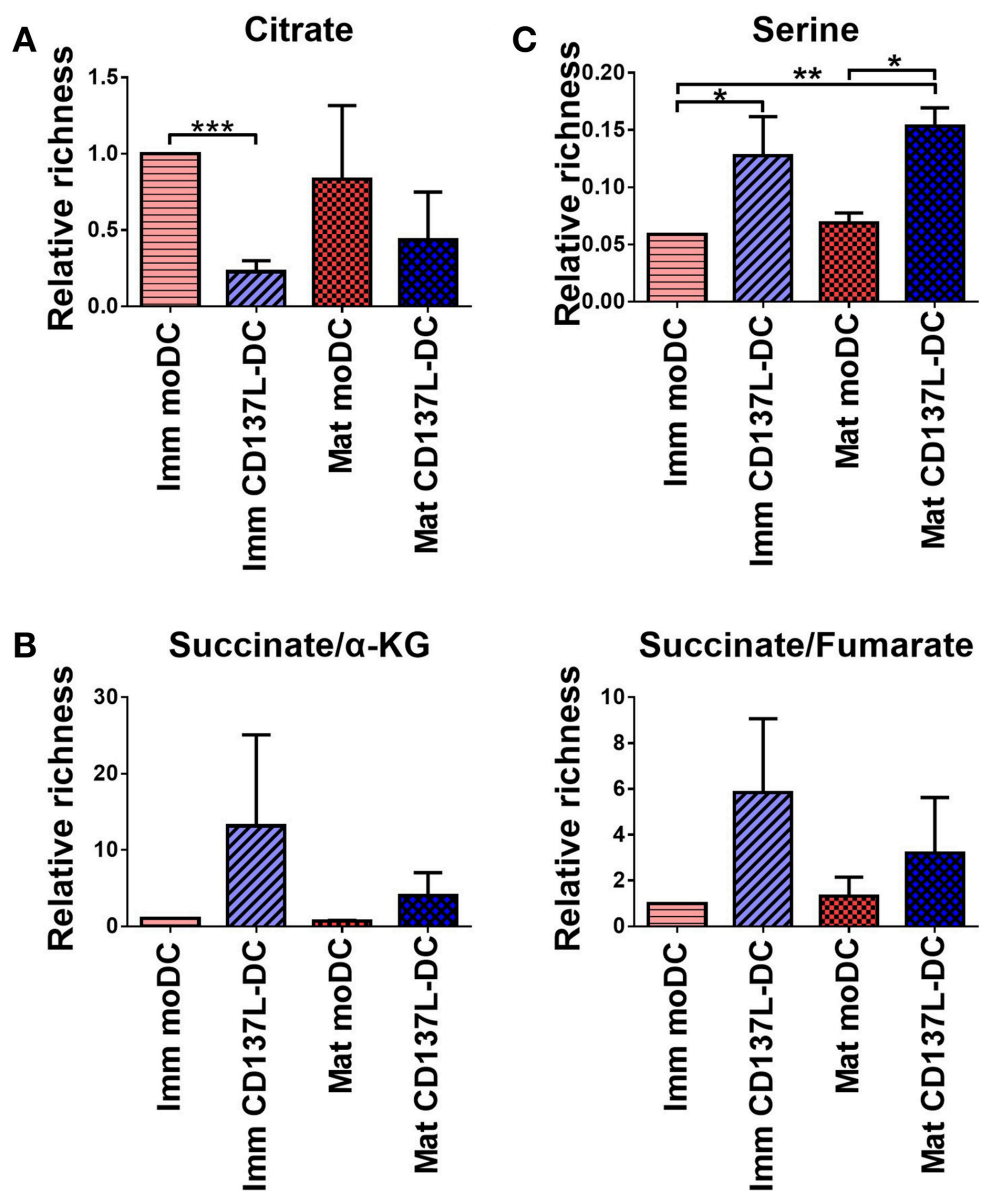
Succinate and serine are enriched in CD137L-DCs. Results from three different donors were normalized based on protein concentrations and statistically analyzed. **(A)** Relative citrate concentrations. **(B)** Relative succinate/α-ketoglutarate and succinate/fumarate ratios. **(C)** Relative abundance of serine. ^*^p < 0.05, ^**^p < 0.01, ^***^p < 0.001 (two-tailed, paired *t* test).

To determine the consequence of the higher glycolysis rate in CD137L-DCs, an unbiased metabolomics experiment, covering amino acids and intermediates from glycolysis and the TCA cycle, was performed. Unexpectedly, citrate, a TCA intermediate that has been reported to accumulate in activated BMDCs (15), was not elevated in mature moDCs, and was lower in CD137L-DCs than moDCs (Figure 6A). However, succinate, another intermediate in TCA cycle, was found to be highly enriched in CD137L-DCs (Figure 6B). A further highly enriched metabolite in CD137L-DCs was serine (Figure 6C), which can be derived from glycolysis. Both succinate and serine play a role in DNA and histone methylation (30, 31).

## Discussion

**Figure 7:**
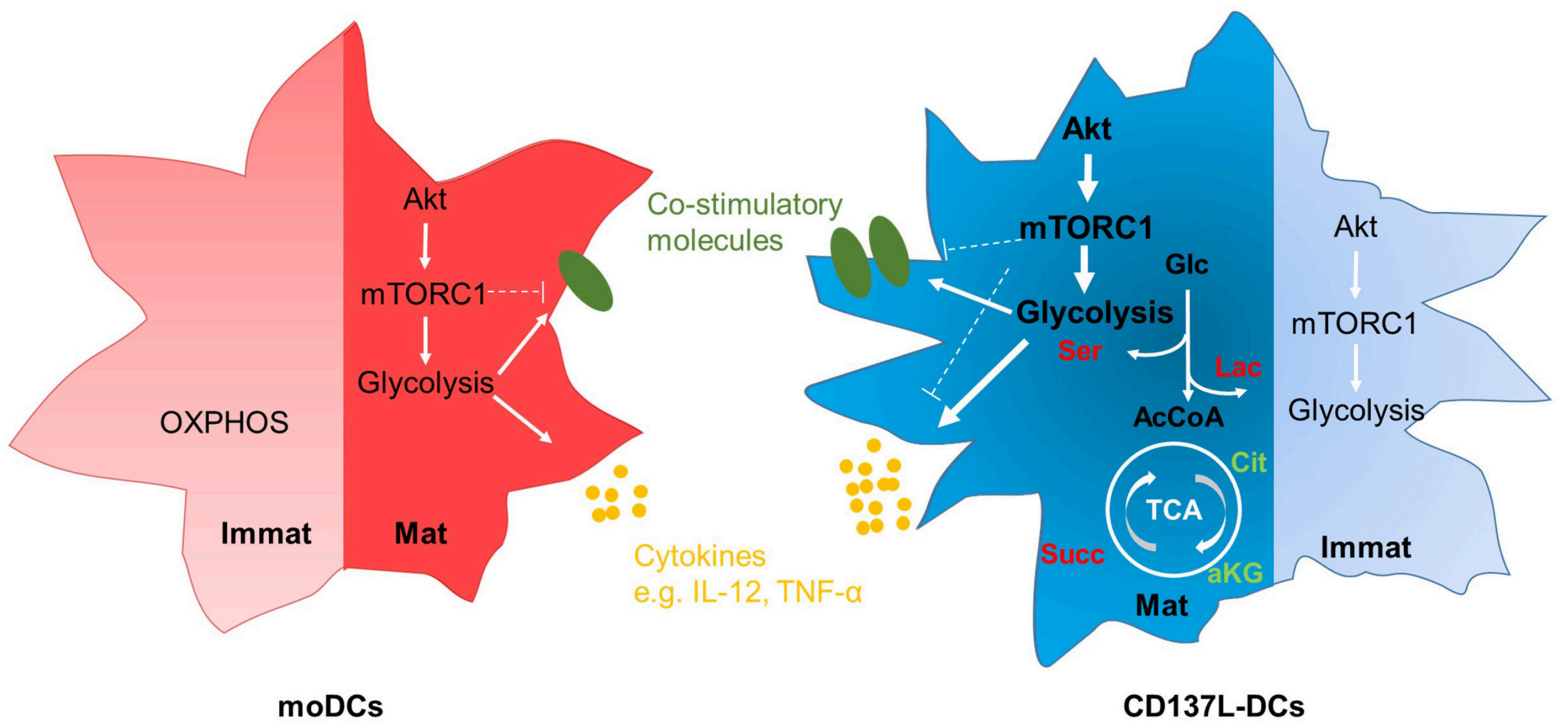
Graphic abstract of the main findings. Immature moDCs have minimal activation of the Akt-mTORC1 pathway, and rely mainly on OXPHOS at the resting stage. In contrast, immature CD137L-DCs have a high activation of the Akt-mTORC1 pathway at the resting stage, leading to an increased glycolysis. After maturation, both mature moDCs and mature CD137L-DCs display an elevated activity of Akt-mTORC1, leading to higher glycolysis and the increased expression of co-stimulatory molecules and pro-inflammatory cytokines. Compared with mature moDCs, mature CD137L-DCs have a significantly higher Akt-driven glycolysis, and secrete more pro-inflammatory cytokines. This higher glycolysis leads to a relative accumulation of succinate and serine rather than citrate or lipids. Red: relative accumulation. Green: relative depletion.

It is increasingly appreciated that metabolic reprograming accompanies the activation of leukocytes. We found that CD137L-DCs have a higher basal glycolysis rate than moDCs because of a higher activity of Akt. After maturation by IFN-γ and the TLR7/8 ligand R848, mature CD137L-DCs have an even higher activity of the Akt-mTORC1 pathway, leading to a further increase in the basal glycolysis rate and the glycolytic capacity. We demonstrated that glycolysis is not only important for the increased expression of co-stimulatory molecules and the increased secretion of inflammatory cytokines during the maturation of CD137L-DCs, but is also important for the preservation of their activated state after maturation. The inhibition of Akt nicely recapitulates the suppressive effects of inhibition of glycolysis on CD137L-DC activity. It is therefore the high Akt-driven glycolysis rate that is the basis for the higher potency of CD137L-DCs compared to moDCs. The essence of our findings is graphically depicted in Figure 7.

Fast growing tumor cells often deplete glucose in the microenvironment (32, 33), leading to a dampened immune response (34). Similarly as tumor cells, T cells upon activation also switch to aerobic glycolysis to support their proliferation (10). In the lymph node, where T cells become primed and activated, glucose level may be low. It is possible that T cells, after being activated by tumor associated antigen (TAA)-loaded DCs, proliferate for some time before the low glucose level in the lymph node suppresses DCs and limits T cell activation. Therefore, multiple injections of *in vitro*-generated DCs are needed to achieve sufficient T cell activation against tumors (35). One advantage of CD137L-DCs is that they are more resistant to spontaneous apoptosis and 2-DG-induced cell death. It is possible that CD137L-DCs survive longer in the lymph node, and therefore deliver stronger and longer-lasting activation to T cells. The plasticity of CD137L-DCs allows them to adapt to the changing environment, and may make it possible to fine-tune the tumor microenvironment and lymph node microenvironment with drugs in order to augment DC-based immunotherapy (36).

We have proven that both Akt and its downstream target mTORC1 mediate the increase of glycolysis in mature CD137L-DCs. However, only the inhibition of Akt during maturation suppresses inflammatory mature CD137L-DCs. The inhibition of mTORC1 by Rapamycin generally enhances the inflammatory features of mature moDCs and mature CD137L-DCs. This discrepancy suggests there are other regulating factors downstream of Akt and mTORC1 besides glycolysis that are involved in the activation of DCs. On top of that, the inhibition of mTORC1 by Rapamycin can be both pro-inflammatory and anti-inflammatory. Sukhbaatar et al. proposed a model where the effect of mTORC1 inhibition on DC function is spatiotemporal: mTORC1 inhibition during early DC activation in the periphery suppresses inflammatory DCs while mTORC1 inhibition during late DC activation in the lymph node enhances the T cell activating ability (37). Our results support this model. For example, the early cytokine IL-1β secreted by mature CD137L-DCs is inhibited, whereas the late cytokine IL-12 is enhanced by Rapamycin.

During the differentiation of DCs, mTORC1 rather than Akt plays the more important role. mTORC1 inhibition blocks the DC differentiation and leads to massive cell death. This blockade of Akt or mTORC1 during differentiation has long-lasting consequences on the generated cells. Even when the monocytes were treated with the PI3K inhibitor LY294002 only during the first 24 h of differentiation, IL-12 secretion was still suppressed in the resulting matured DC on day 7. But the opposite, i.e., and enhancement of IL-12 secretion in resulting DC, was obtained when monocytes had been treated with the mTOR inhibitor Rapamycin. This long-term effect resembles the reported innate memory where monocytes are more inflammatory to a second stimulus (38). The molecular basis for this long-term effect may be the Akt-mTORC1-mediated glycolysis which has been reported to be involved in the epigenetic regulation of monocyte memory (39).

Everts and colleagues suggested that the increased glycolysis during activation results in an accumulation of citrate for the synthesis of lipids, which expands the ER and Golgi apparatus (15). However, we could not find an accumulation of citrate. Neither did we observe a higher lipid content in moDCs or CD137L-DCs after maturation, nor could we measure more lipids in CD137L-DCs than in moDCs. It has been shown that in monocytes different TLR ligands induce very different metabolic changes and transcriptomes (40). It is possible that the increased lipid synthesis is specific to LPS-activated moDCs but not to CD137L-DCs or cytokine-activated moDCs. The two fatty acid synthesis inhibitors, C75 and TOFA, did not decrease the lipid contents in any of the four types of DCs. However, the inhibition of FASN by C75 inhibited the maturation of both, CD137L-DCs and moDCs. It is possible that C75 reduces the level of Acetyl-CoA for acetylation, which plays an important role in the regulation of inflammation-related gene expression (41). Another possibility is that C75 alters the ratio of pro-inflammatory lipids to anti-inflammatory lipids (42).

Succinate and α-ketoglutarate have been reported to be involved in the polarization of M1 and M2 macrophages (43). Succinate accumulates in M1 macrophages and promotes inflammation (44). A higher succinate to α-ketoglutarate ratio preferentially induces pro-inflammatory macrophage differentiation while a lower succinate / α-ketoglutarate ratio promotes anti-inflammatory macrophage differentiation (31). It is very likely that the higher succinate/α-ketoglutarate ratio contributes to the pro-inflammatory features of CD137L-DC.

Succinate and α-ketoglutarate are also involved in the epigenetic regulation of cancer cells and macrophages (31, 45). Serine as an indispensable substrate for the synthesis of S-adenosylmethionine (SAM), a methyl group donor, plays a role in the epigenetic regulation of gene expression (30). The accumulation of succinate and serine in CD137L-DC might not be a coincidence, but may have a synergistic effect on epigenetic upregulation of pro-inflammatory gene expression.

Inflammatory DCs, M1 macrophages and effector T cells all reprogram their metabolism and increase glycolysis rates upon activation (46), and the function of these cells can be dampened if glycolysis is inhibited. However, it is unknown at present if the functions of these cells can be enhanced by simply increasing their (1) glycolysis, (2) activation of the Akt-mTORC1 pathway, or (3) their ability to compete for glucose in the tumor microenvironment. We have tried to achieve this by using the Akt agonist SC-79 (47) and the mTORC agonist MHY1485 (48), but to no avail. It is also not clear by what mechanisms glycolysis supports the functions of these immune cells. Our data argue for further in-depth investigation of the already increasingly appreciated interplay between metabolism and epigenetics.

It would have been informative to demonstrate the enhanced Akt-driven glycolysis as the basis of the higher potency *in vivo*, e.g., in a murine tumor model, by genetic manipulation of key glycolytic enzymes (e.g., HK2) specifically in CD137L-DCs. Unfortunately, CD137L-DCs do not exist in mouse (7), and the reason may be the large difference in CD137L between human and mouse. While for most members of the TNF and TNF receptor families the human—mouse homology is 60–80%, it is only 36% for human and murine CD137L (49).

In summary, we have demonstrated (1) that the Akt-driven glycolysis is crucial for the sustained activation of CD137L-DCs, (2) that the higher Akt-driven glycolysis is part of the reason why CD137L-DCs are more potent than the conventional moDCs, and (3) that Akt-driven glycolysis leads to an accumulation of succinate and serine instead of lipids in CD137L-DCs. Our finding suggests that the Akt-driven glycolysis could be a therapeutic target to manipulate the function of CD137L-DCs for better clinical efficacy.

## Ethics Statement

Blood was obtained from healthy volunteers who provided written and informed consent. The protocol was approved by the National University of Singapore (NUS) IRB number B15-320E.

## Author Contributions

QZ and HS: conceptualization and writing—original draft; QZ and KM: methodology; QZ: investigation and data curation; HS, QZ, and KM: writing—review and editing; HS: supervision and funding acquisition.

### Conflict of Interest Statement

The authors declare that the research was conducted in the absence of any commercial or financial relationships that could be construed as a potential conflict of interest.

## Abbreviations

ACC: acetyl-CoA carboxylase
BMDCs: bone marrow-derived DCs
CD137L-DC: CD137 ligand-induced DC
DC: Dendritic cell
FASN: fatty acid synthase
moDC: monocyte-derived DC
OXPHOS: oxidative phosphorylation.
