# Supplementary material for: Increased Akt-Driven Glycolysis Is the Basis for the Higher Potency of CD137L-DCs
Source: Front Immunol. 2019 Apr 24;10:868. doi: 10.3389/fimmu.2019.00868 (PMC6491642; doi:10.3389/fimmu.2019.00868)
Supplement: Supplementary file 1 [file Presentation_1.pptx]

## Slide 1
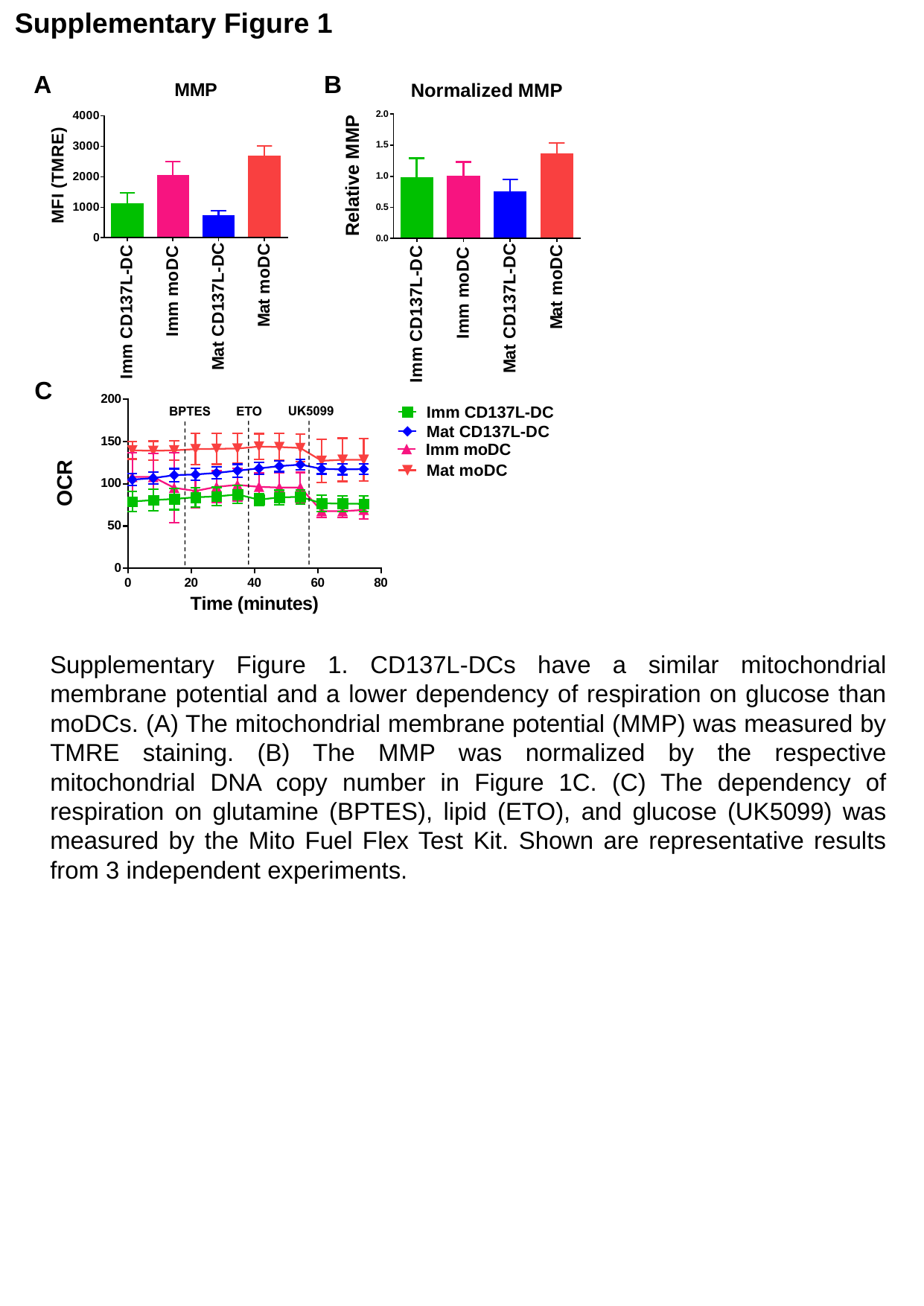

Supplementary Figure 1
A
B
C
Supplementary Figure 1. CD137L-DCs have a similar mitochondrial membrane potential and a lower dependency of respiration on glucose than moDCs. (A) The mitochondrial membrane potential (MMP) was measured by TMRE staining. (B) The MMP was normalized by the respective mitochondrial DNA copy number in Figure 1C. (C) The dependency of respiration on glutamine (BPTES), lipid (ETO), and glucose (UK5099) was measured by the Mito Fuel Flex Test Kit. Shown are representative results from 3 independent experiments.

## Slide 2
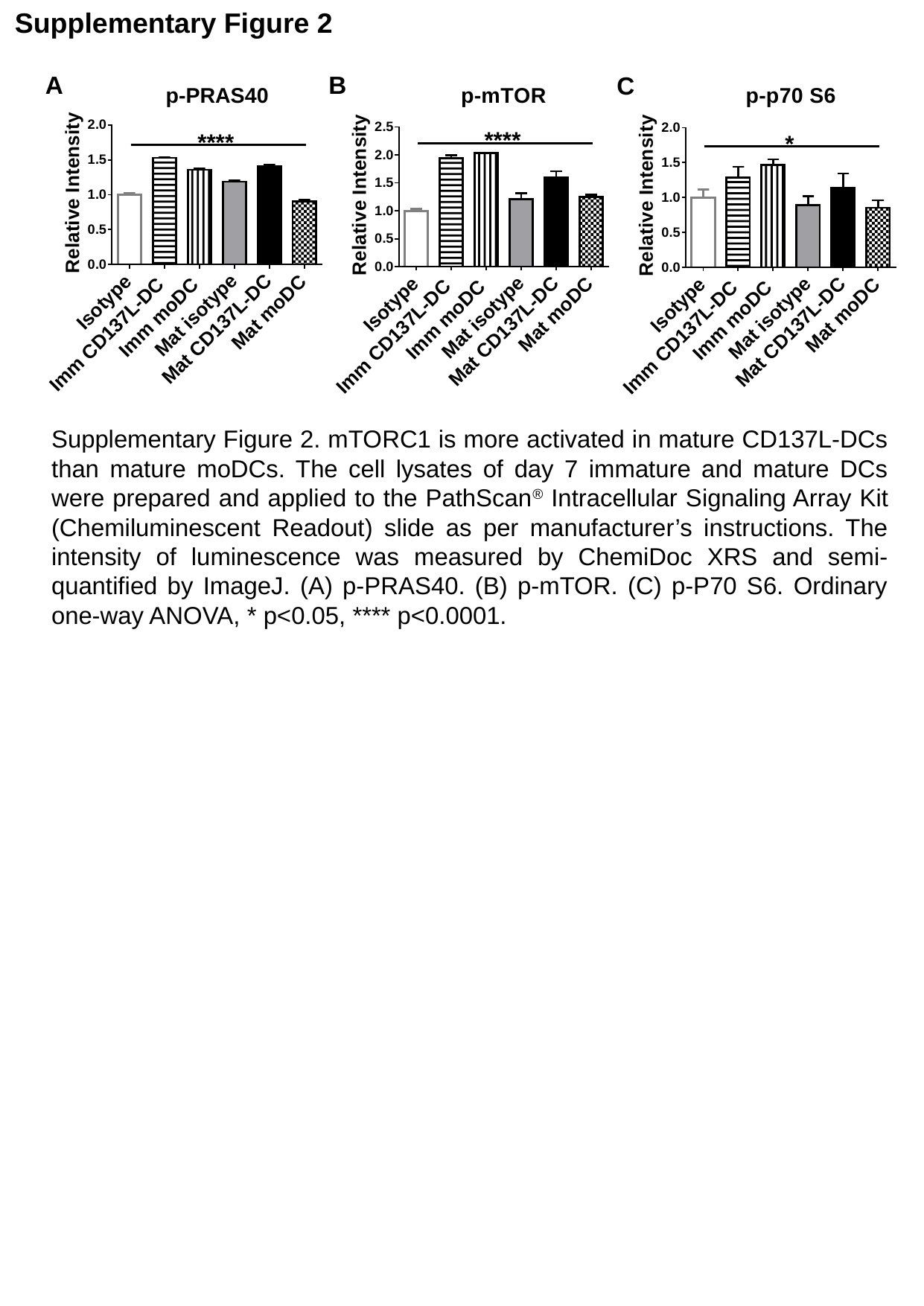

Supplementary Figure 2
B
A
C
Supplementary Figure 2. mTORC1 is more activated in mature CD137L-DCs than mature moDCs. The cell lysates of day 7 immature and mature DCs were prepared and applied to the PathScan® Intracellular Signaling Array Kit (Chemiluminescent Readout) slide as per manufacturer’s instructions. The intensity of luminescence was measured by ChemiDoc XRS and semi-quantified by ImageJ. (A) p-PRAS40. (B) p-mTOR. (C) p-P70 S6. Ordinary one-way ANOVA, * p<0.05, **** p<0.0001.

## Slide 3
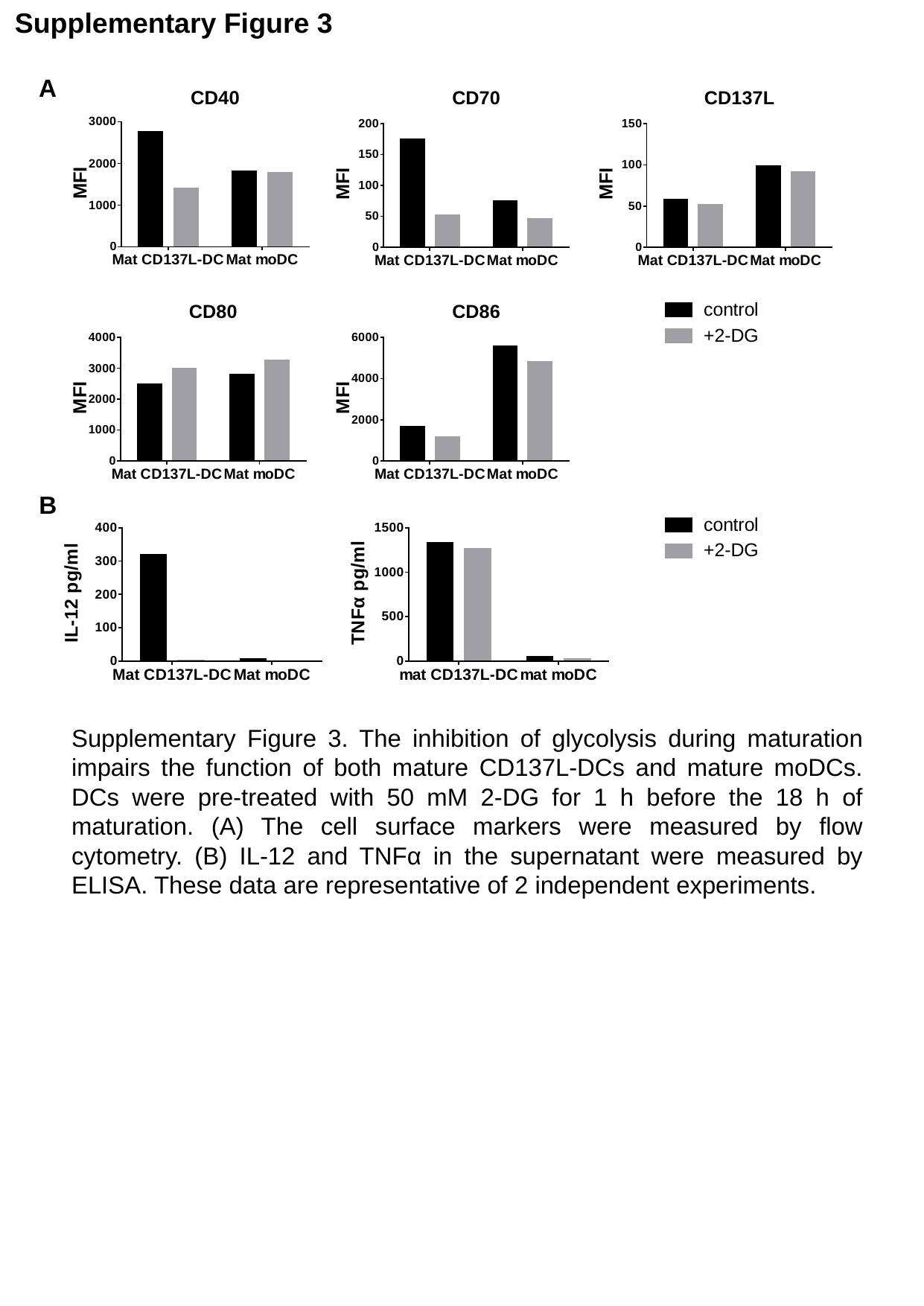

Supplementary Figure 3
A
B
Supplementary Figure 3. The inhibition of glycolysis during maturation impairs the function of both mature CD137L-DCs and mature moDCs. DCs were pre-treated with 50 mM 2-DG for 1 h before the 18 h of maturation. (A) The cell surface markers were measured by flow cytometry. (B) IL-12 and TNFα in the supernatant were measured by ELISA. These data are representative of 2 independent experiments.

## Slide 4
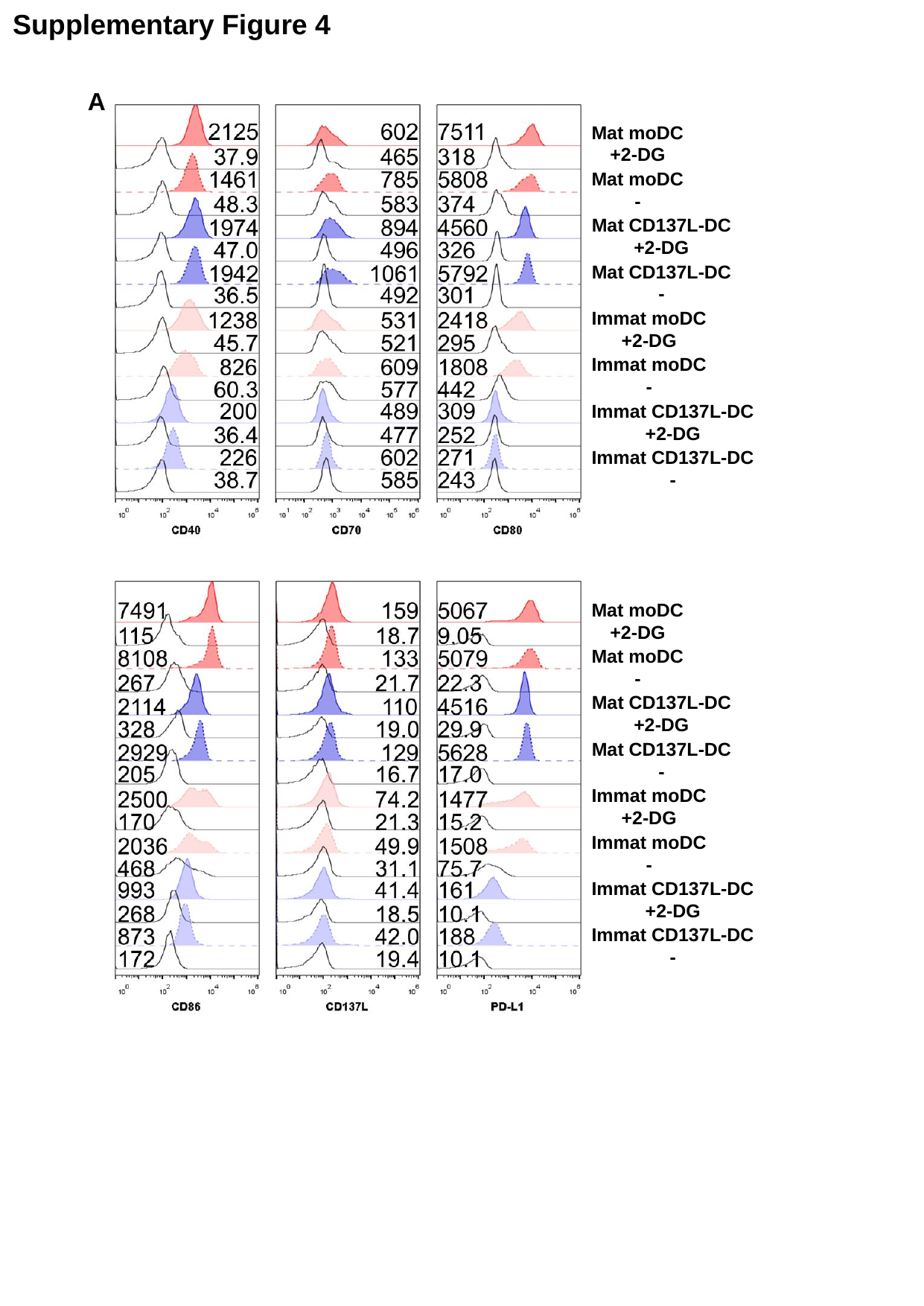

Supplementary Figure 4
Mat moDC
+2-DG
Mat moDC
-
Mat CD137L-DC
+2-DG
Mat CD137L-DC
-
Immat moDC
+2-DG
Immat moDC
-
Immat CD137L-DC
+2-DG
Immat CD137L-DC
-
Mat moDC
+2-DG
Mat moDC
-
Mat CD137L-DC
+2-DG
Mat CD137L-DC
-
Immat moDC
+2-DG
Immat moDC
-
Immat CD137L-DC
+2-DG
Immat CD137L-DC
-
A

## Slide 5
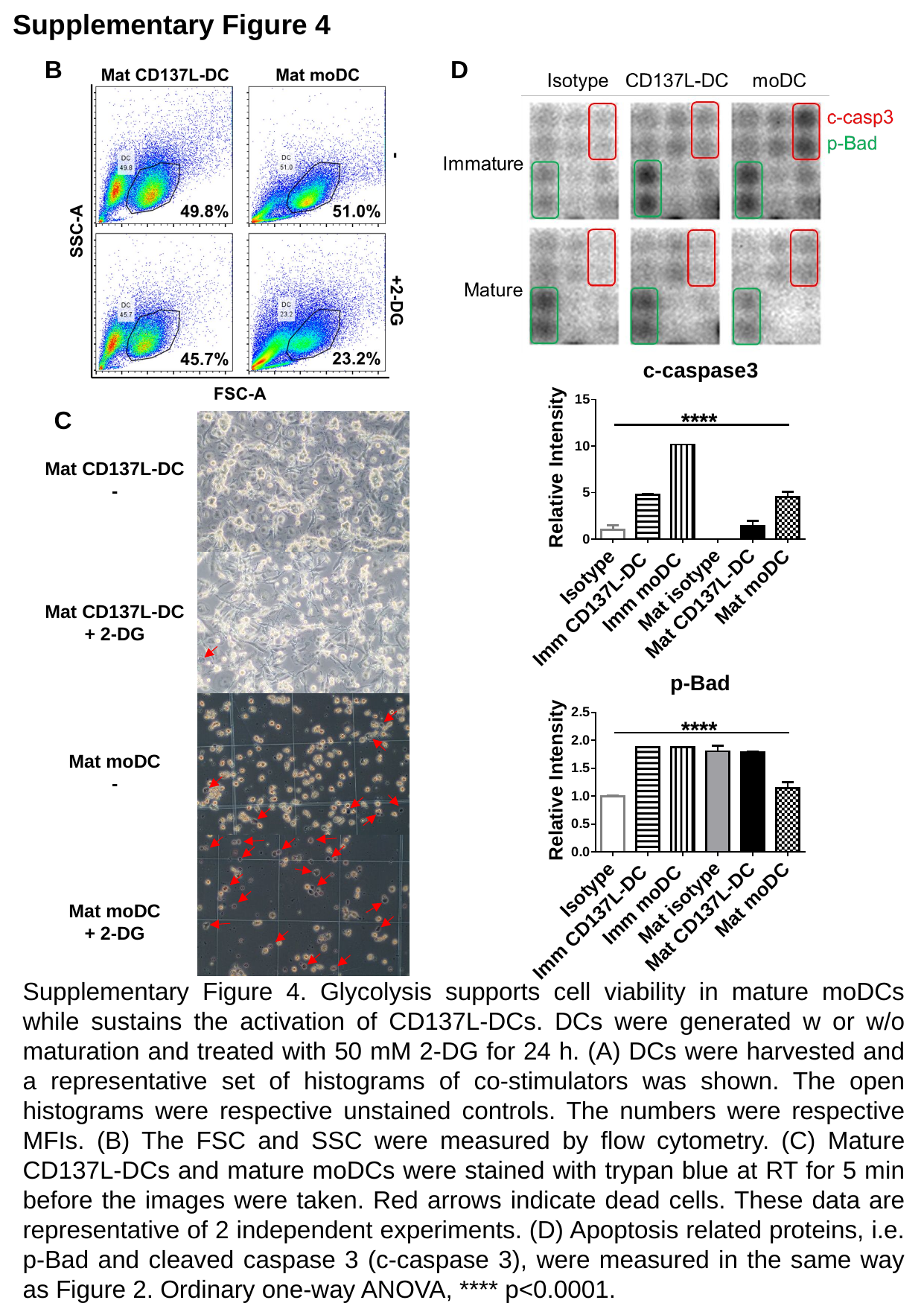

Supplementary Figure 4
B
D
C
Mat CD137L-DC
-
Mat CD137L-DC
+ 2-DG
Mat moDC
-
Mat moDC
+ 2-DG
Supplementary Figure 4. Glycolysis supports cell viability in mature moDCs while sustains the activation of CD137L-DCs. DCs were generated w or w/o maturation and treated with 50 mM 2-DG for 24 h. (A) DCs were harvested and a representative set of histograms of co-stimulators was shown. The open histograms were respective unstained controls. The numbers were respective MFIs. (B) The FSC and SSC were measured by flow cytometry. (C) Mature CD137L-DCs and mature moDCs were stained with trypan blue at RT for 5 min before the images were taken. Red arrows indicate dead cells. These data are representative of 2 independent experiments. (D) Apoptosis related proteins, i.e. p-Bad and cleaved caspase 3 (c-caspase 3), were measured in the same way as Figure 2. Ordinary one-way ANOVA, **** p<0.0001.

## Slide 6
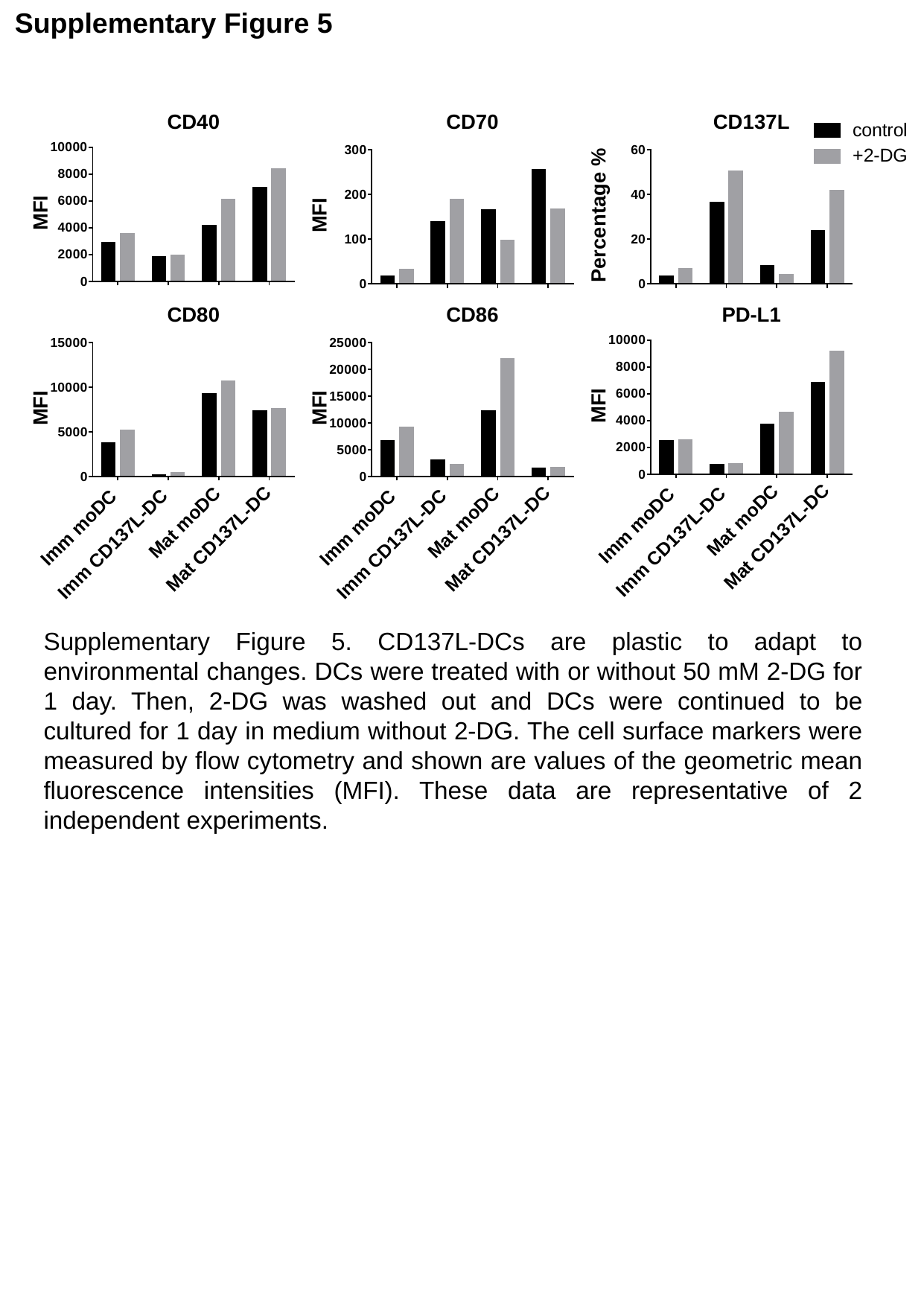

Supplementary Figure 5
Supplementary Figure 5. CD137L-DCs are plastic to adapt to environmental changes. DCs were treated with or without 50 mM 2-DG for 1 day. Then, 2-DG was washed out and DCs were continued to be cultured for 1 day in medium without 2-DG. The cell surface markers were measured by flow cytometry and shown are values of the geometric mean fluorescence intensities (MFI). These data are representative of 2 independent experiments.

## Slide 7
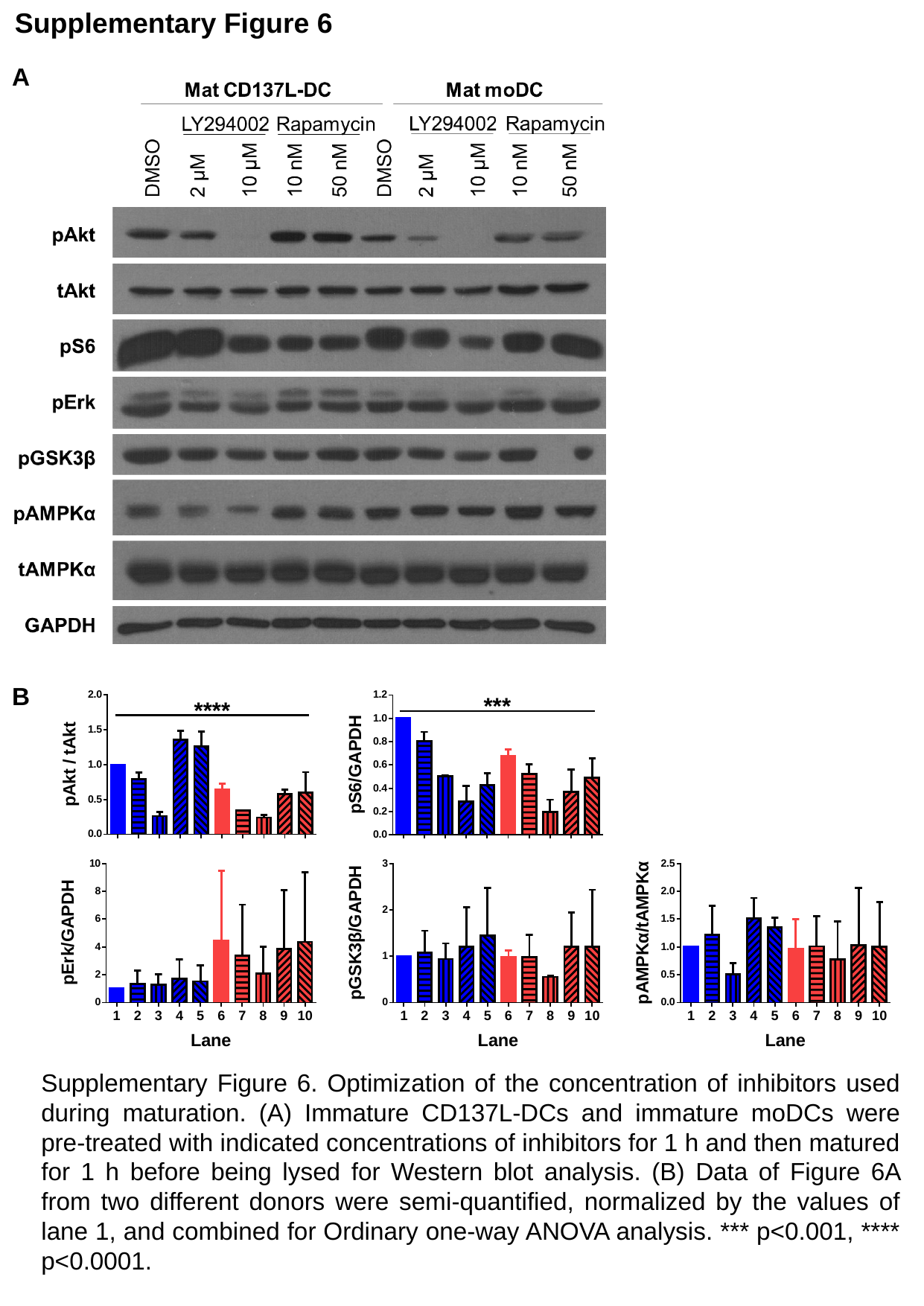

Supplementary Figure 6
A
B
Supplementary Figure 6. Optimization of the concentration of inhibitors used during maturation. (A) Immature CD137L-DCs and immature moDCs were pre-treated with indicated concentrations of inhibitors for 1 h and then matured for 1 h before being lysed for Western blot analysis. (B) Data of Figure 6A from two different donors were semi-quantified, normalized by the values of lane 1, and combined for Ordinary one-way ANOVA analysis. *** p<0.001, **** p<0.0001.

## Slide 8
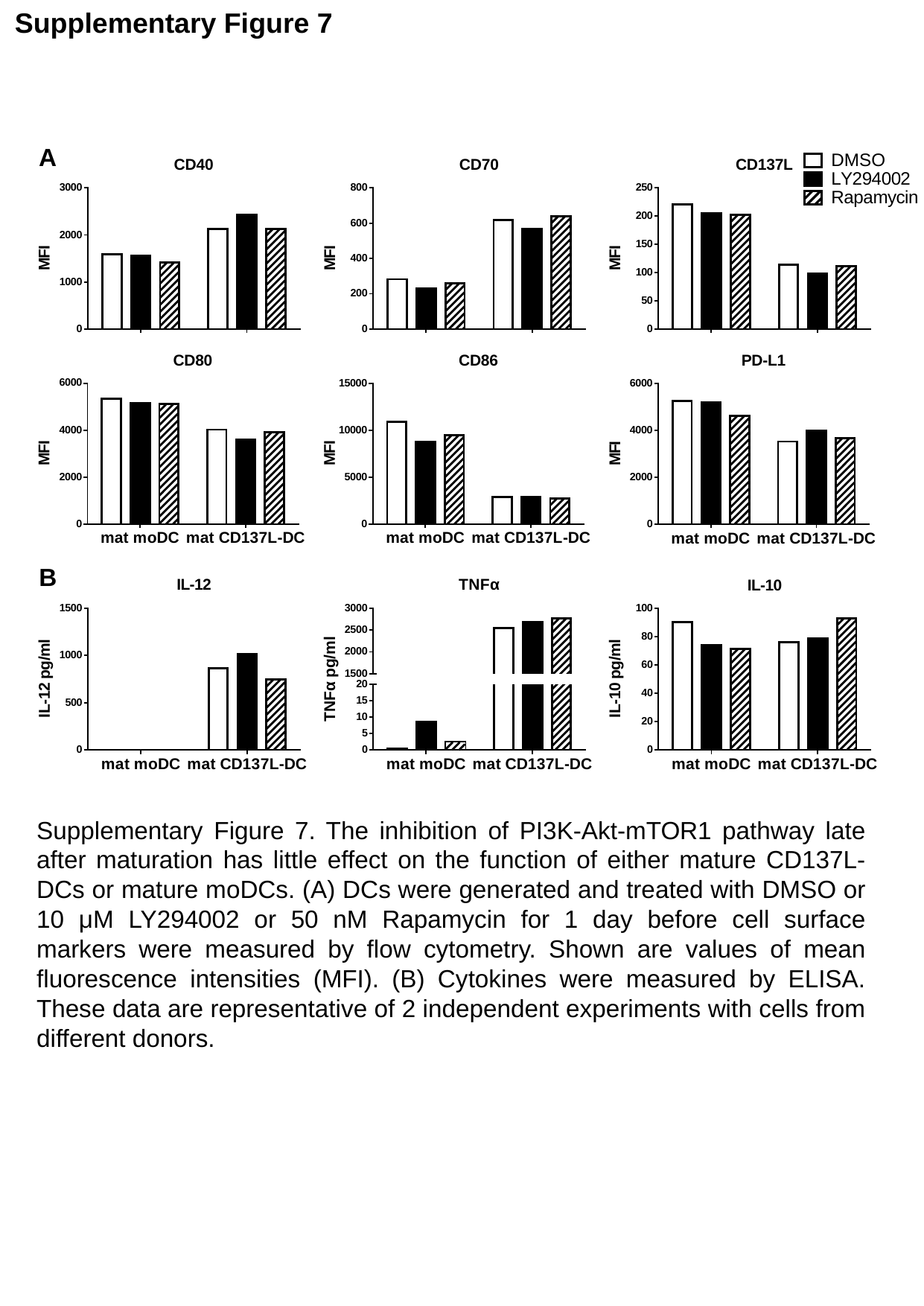

Supplementary Figure 7
A
B
Supplementary Figure 7. The inhibition of PI3K-Akt-mTOR1 pathway late after maturation has little effect on the function of either mature CD137L-DCs or mature moDCs. (A) DCs were generated and treated with DMSO or 10 μM LY294002 or 50 nM Rapamycin for 1 day before cell surface markers were measured by flow cytometry. Shown are values of mean fluorescence intensities (MFI). (B) Cytokines were measured by ELISA. These data are representative of 2 independent experiments with cells from different donors.

## Slide 9
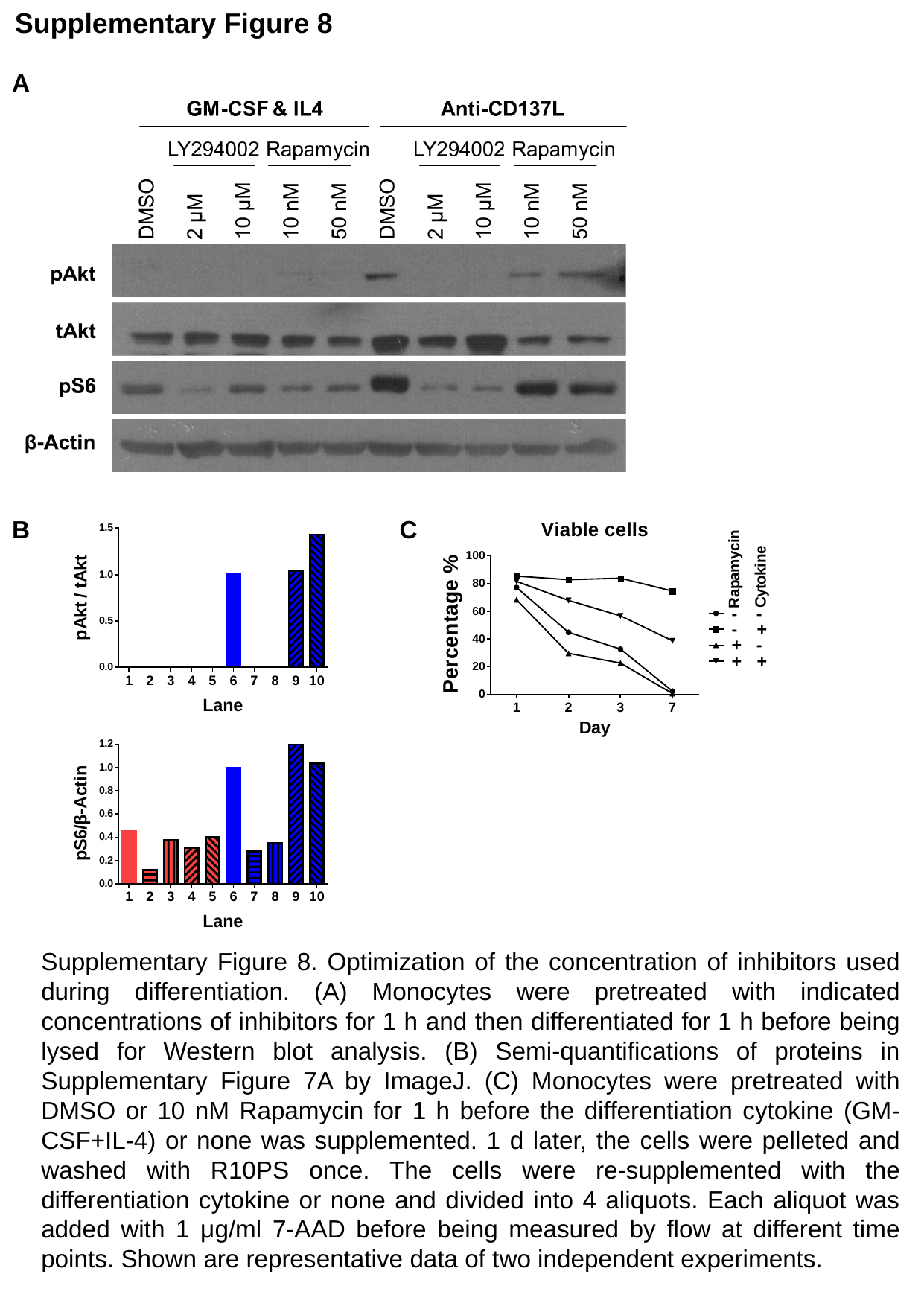

Supplementary Figure 8
A
B
C
Supplementary Figure 8. Optimization of the concentration of inhibitors used during differentiation. (A) Monocytes were pretreated with indicated concentrations of inhibitors for 1 h and then differentiated for 1 h before being lysed for Western blot analysis. (B) Semi-quantifications of proteins in Supplementary Figure 7A by ImageJ. (C) Monocytes were pretreated with DMSO or 10 nM Rapamycin for 1 h before the differentiation cytokine (GM-CSF+IL-4) or none was supplemented. 1 d later, the cells were pelleted and washed with R10PS once. The cells were re-supplemented with the differentiation cytokine or none and divided into 4 aliquots. Each aliquot was added with 1 μg/ml 7-AAD before being measured by flow at different time points. Shown are representative data of two independent experiments.

## Slide 10
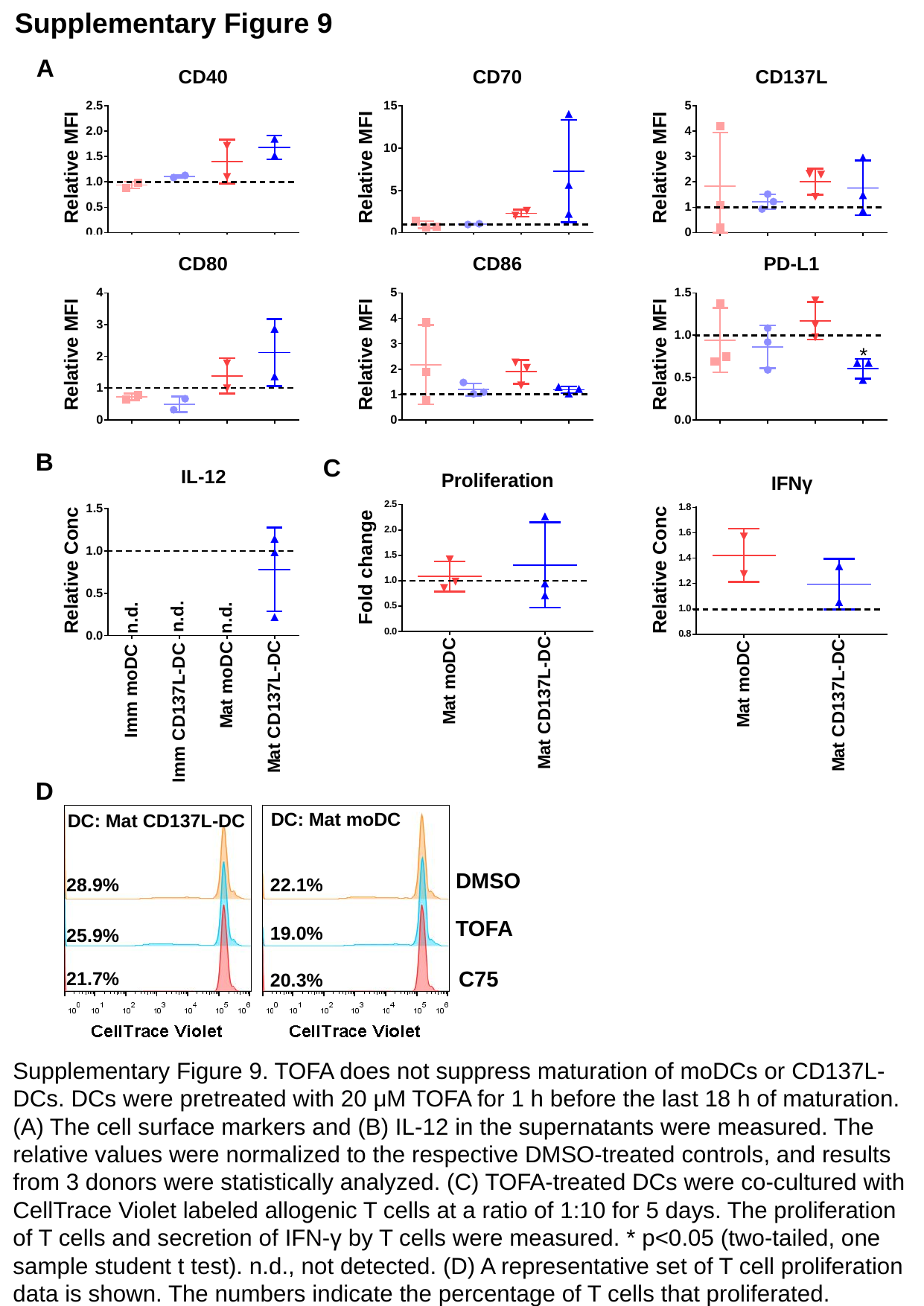

Supplementary Figure 9
A
B
C
D
DC: Mat moDC
DC: Mat CD137L-DC
28.9%
22.1%
19.0%
25.9%
21.7%
20.3%
DMSO
TOFA
C75
Supplementary Figure 9. TOFA does not suppress maturation of moDCs or CD137L-DCs. DCs were pretreated with 20 μM TOFA for 1 h before the last 18 h of maturation. (A) The cell surface markers and (B) IL-12 in the supernatants were measured. The relative values were normalized to the respective DMSO-treated controls, and results from 3 donors were statistically analyzed. (C) TOFA-treated DCs were co-cultured with CellTrace Violet labeled allogenic T cells at a ratio of 1:10 for 5 days. The proliferation of T cells and secretion of IFN-γ by T cells were measured. * p<0.05 (two-tailed, one sample student t test). n.d., not detected. (D) A representative set of T cell proliferation data is shown. The numbers indicate the percentage of T cells that proliferated.
